# Time-series metatranscriptomics reveals differential salinity effects on the methanogenic food web in paddy soil

**DOI:** 10.1128/msystems.00017-25

**Published:** 2025-07-21

**Authors:** Xi Zhou, Xin Li, Qicheng Bei, Xingjie Wu, Guihua Xu, Xiuzhu Dong, Werner Liesack, Zhenling Cui, Fusuo Zhang, Jingjing Peng

**Affiliations:** 1 State Key Laboratory of Nutrient Use and Management, College of Resources and Environmental Sciences, National Academy of Agriculture Green Development, China Agricultural University 34752, Beijing, China; 2 State Key Laboratory of Efficient Utilization of Arid and Semi-arid Arable Land in Northern China, Beijing, China; 3 Institute of Agricultural and Nutritional Sciences, Martin-Luther-Universität Halle Wittenberg 153730, Halle (Saale), Germany; 4 Department of Biological Sciences, University of Southern California 5116, Los Angeles, California, USA; 5 School of Light Industry Science and Engineering, Beijing Technology and Business University 58276, Beijing, China; 6 State Key Laboratory of Microbial Resources, Institute of Microbiology, Chinese Academy of Sciences 85387, Beijing, China; 7 Max Planck Institute for Terrestrial Microbiology 28310, Marburg, Germany; University of Wisconsin-Milwaukee, Milwaukee, Wisconsin, USA

**Keywords:** CH_4_, methanogenesis, CAZyme, metatranscriptomics, salt stress

## Abstract

Saltwater intrusion and sea level rise (SWISLR) threaten coastal agroecosystems, yet their impact on the methanogenic food web in rice paddies remains virtually unknown. Using “double-RNA” metatranscriptomics (rRNA, mRNA), we investigated salinity effects on the methanogenic community in straw-amended slurries following different preincubation periods. Our results demonstrate that salt stress significantly inhibits methane (CH_4_) production, with the degree of inhibition varying by the community’s successional stage. Salinity fundamentally shifted the methanogenic food web toward *Clostridiaceae* dominance, altering key metabolic pathways including polymer breakdown, glycolysis, pyruvate metabolism, decarboxylation conversion of pyruvate to acetate, and ethanol fermentation. Notably, the accumulation of acetate under salt stress fueled acetoclastic methanogenesis by *Methanosarcinaceae*, mitigating CH_4_ production inhibition after a 14-day preincubation. While the strong salinity inhibitory effect on the expression of the hydrogenotrophic methanogenesis pathway by *Methanocellaceae* diminished with preincubation time, syntrophic propionate oxidation activity was highly suppressed after 14-day preincubation. Additionally, the expression of the methanol-dependent methanogenesis pathway by *Methanomassiliicoccales* increased with prolonged preincubation time. These findings provide critical insights into the resilience of the anaerobic food web under salinity stress, with broader implications for understanding the impacts of sea level rise on global biogeochemical cycles.

**IMPORTANCE:**

Seawater intrusion and sea level rise (SWISLR), driven by climate change, pose significant threats to coastal agroecosystems, particularly salt-affected paddy soils. Despite the importance of these systems in global methane dynamics, the specific effects of salinity on the methanogenic food web in rice paddies remain poorly understood. Using a “double-RNA” metatranscriptomics approach, this study demonstrates that salinity markedly alters methane production and microbial community dynamics, with these effects varying across different successional stages of the microbial assemblage. The resilience of the methanogenic food web under salinity stress is governed by time-dependent metabolic shifts, offering critical insights into how SWISLR may influence methane emissions and broader biogeochemical processes in coastal agricultural landscapes. These findings highlight the urgent need to incorporate SWISLR-related impacts into assessments of coastal agroecosystems’ contributions to global methane budgets and climate feedback mechanisms.

## INTRODUCTION

Saltwater intrusion and sea level rise (SWISLR) driven by climate change pose significant threats to coastal agroecosystems, particularly salt-affected paddy soils, which are critical global carbon stocks and sources of CH_4_ emissions (1–3). Understanding these impacts is critical because more than 20% of the world’s rice paddies, especially those in coastal and delta regions, are already affected by salt stress (4, 5). These soils support diverse microbial communities but are highly susceptible to environmental stressors, such as salinity, which can influence their capacity as carbon sinks (2, 6).

The methanogenic food web in paddy soils plays a central role in the decomposition of organic matter, primarily rice straw, by hydrolytic, fermenting, and syntrophic bacteria, as well as methanogens, leading to CH_4_ and CO_2_ production (7–12). Hydrolytic bacteria break down complex plant polymers into simpler oligomers and monomers, which are subsequently fermented to short-chain fatty acids and alcohols, fueling methanogenesis via acetate, H_2_–CO_2_, and methylated compounds (13, 14). Acetoclastic and hydrogenotrophic pathways are dominant at a ratio of 2:1 in rice paddies (12, 15). The methanogenic community can be dramatically affected by various environmental factors, such as temperature (14, 16), freeze-thaw cycles (17), dry-wet transitions (18), nitrogen level (19), and the soil type (20), while few studies have examined how SWISLR and salinity impact microbial activity and gene expression within the methanogenic food web in rice field soils.

To elucidate the short-term impact of increasing salinity on the metabolic activity of particular functional guilds, we conducted a study focusing on different microbial populations and their role within the methanogenic food web, including polymer hydrolysis, fermentation, and methanogenesis. Our experimental setup involved slurry microcosms incubated under anoxic conditions, with the addition of rice straw as a carbon source. The straw-amended microcosms were preincubated without salt stress for 9, 14, 21, and 28 days at 30°C. The 28-day period has been shown to result in a complete consumption of the easily degradable rice straw components (15). This ultimately involves successional changes in the composition and activity of the methanogenic community with progressing polymer breakdown (12, 14). Upon each preincubation time, the microbial communities were exposed to zero (control), moderate (1.75%), and high (3.50%) salinity for 48 hours (Fig. S1). This experimental approach simulates the scenario of SWISLR in paddy lands. We applied a “double-RNA” metatranscriptomic approach as our key methodology to monitor both the structural (16S rRNA) and functional (mRNA) responses of the complex methanogenic community to salt stress (12, 21–23). We aimed to answer the following questions: (i) how do bacteria and methanogens respond to varying salinity levels across different stages of community succession; and (ii) what underlying microbial mechanisms drive salinity-induced dynamics within the anaerobic food web? Although many studies on salt stress in anaerobic environments have focused on methanogens, comparatively less attention has been given to higher trophic levels involving bacterial communities responsible for polymer hydrolysis and fermentation processes. These bacterial guilds play a critical role in determining substrate availability for methanogens. In other ecosystems, taxa such as *Firmicutes and Bacteroidetes* have been reported to possess osmotolerant traits, including solute transporters or stress proteins (24). However, the expression and functional relevance of these traits under SWISLR-like conditions in paddy soils remain poorly characterized. We hypothesized that the community successional stage would have a differential effect on the salt-induced suppression of CH_4_ production, while the key players in carbon metabolism and CH_4_ emission would remain dominant under stress conditions due to their physiological traits.

## RESULTS

### Differential salinity effect on CH_4_ and CO_2_ production

**Fig 1:**
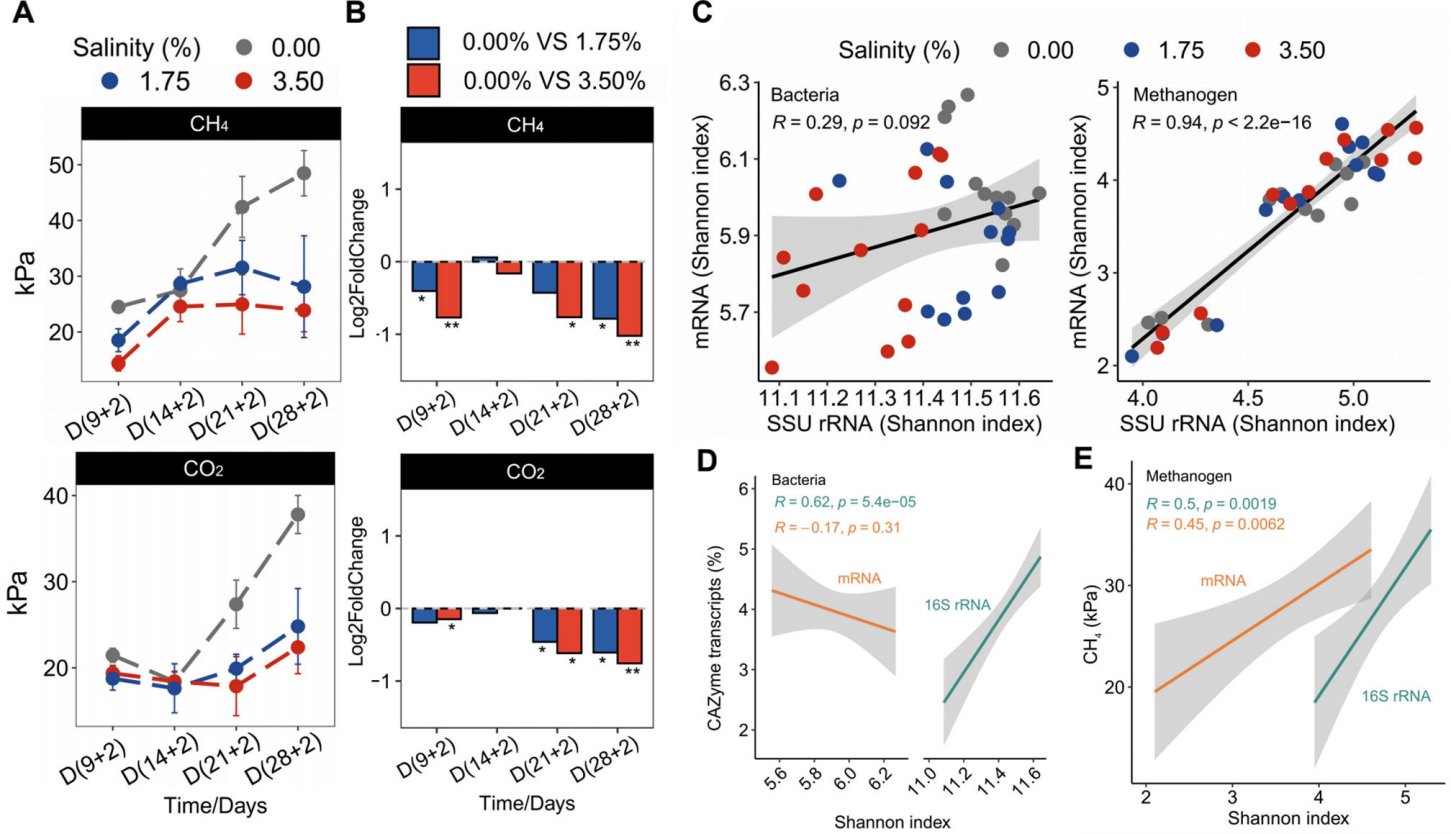
Community-wide activity response to salt stress. (**A**) Line plots display the concentrations of CH_4_ production and CO_2_ evolution in response to salt stress after the four different preincubation time points. Absolute concentrations of CH_4_ and CO_2_ are shown for three salinity levels (0.00%, 1.75%, 3.50%) (mean ± SE, *n* = 3). Please note that the dashed lines connecting the preincubation times are only shown for improved visualization of the overall results. (**B**) Bar plots display the effects of salt stress on CH_4_ production and CO_2_ evolution, shown as log2 fold change relative to the control treatment. Negative values at the different salinity levels indicate an inhibition effect relative to the control. The paired *t*-test was used to assess the statistical significance of differences between treatments. Significance is denoted by asterisks (*P < 0.05, **P < 0.01, ***P < 0.001). (**C**) Correlations between the alpha diversity (Shannon index) of 16S rRNA and mRNA were analyzed for bacteria and methanogens across all the treatments. The colors indicate the different salinity treatments. (**D**) Correlations between the bacterial diversity (Shannon index) and the relative abundance of carbohydrate-active enzyme (CAZyme) transcripts. (**E**) Correlations between the methanogen diversity (Shannon index) and CH_4_ emission. The green and orange colors in (**D**) and (**E**) represent the correlation analysis on the 16S rRNA and mRNA levels, respectively. The “R” (**C–E**) denotes the Pearson correlation coefficient, and the *P-*value is used to test the null hypothesis that there is no linear relationship between the two variables, *n* = 36.

Moderate (1.75%) and high (3.50%) salinity were applied to investigate their effects on the anaerobic food web and CH_4_ production in rice paddies. Both salinity levels inhibited CH_4_ and CO_2_ production relative to the control on days (9 + 2), (21 + 2), and (28 + 2), but not on day (14 + 2) (Fig. 1A and B). Acetate accumulated under salt stress conditions at all four preincubation times, particularly evident on day (14 + 2) (*P* < 0.001). Propionate was significantly enriched with high significance under salt stress at days (14 + 2) and (28 + 2), but not on days (9 + 2) and (21 + 2) (Fig. S2). A significant negative correlation was observed between acetate and CH_4_ production across all four time points (R = −0.63, *P* < 0.001) (Fig. S3).

### Greater salinity effect on bacteria than methanogens

We applied double-RNA metatranscriptomics to determine the microbial response to salt stress. Salt stress significantly affected the bacterial community composition on the mRNA level more than on the 16S rRNA level (*P* < 0.001), while the methanogen community was more affected by the preincubation time than the salt stress treatments (*P* < 0.001) (Fig. S4, Table S3 and S4). The exposure to high salinity, but not to moderate salinity, significantly lowered the bacterial alpha diversity on the 16S rRNA level, as indicated by the Shannon index (*P* < 0.001) (Fig. S5). By contrast, the alpha diversity of methanogens varied significantly with preincubation time on both the 16S rRNA level (time: *P* < 0.001; salinity: *P* = 0.10, adonis test) and the mRNA level (time: *P* < 0.001; salinity: *P* = 0.11, adonis test) (Fig. S5).

The alpha diversity of methanogens showed a significant positive correlation between the 16S rRNA and mRNA levels (R = 0.94, *P* < 0.001), while such a correlation was not evident within the bacterial community (Fig. 1C). The relative abundance of carbohydrate-active enzyme (CAZyme) transcripts was positively correlated with the bacterial alpha diversity on the 16S rRNA level (R = 0.62, *P* < 0.001), but not on the mRNA level (Fig. 1D). Furthermore, a significant positive correlation was observed between the alpha diversity of methanogens and CH_4_ production on both the 16S rRNA and the mRNA levels (*P* < 0.01) (Fig. 1E). The alpha diversity of mRNA encoding polymer breakdown (CAZymes) and methane metabolism also showed a significant positive correlation with CH_4_ production (*P* < 0.001) (Fig. S3 and S5).

### Distinct microbial community responses to increasing salt stress

**Fig 2:**
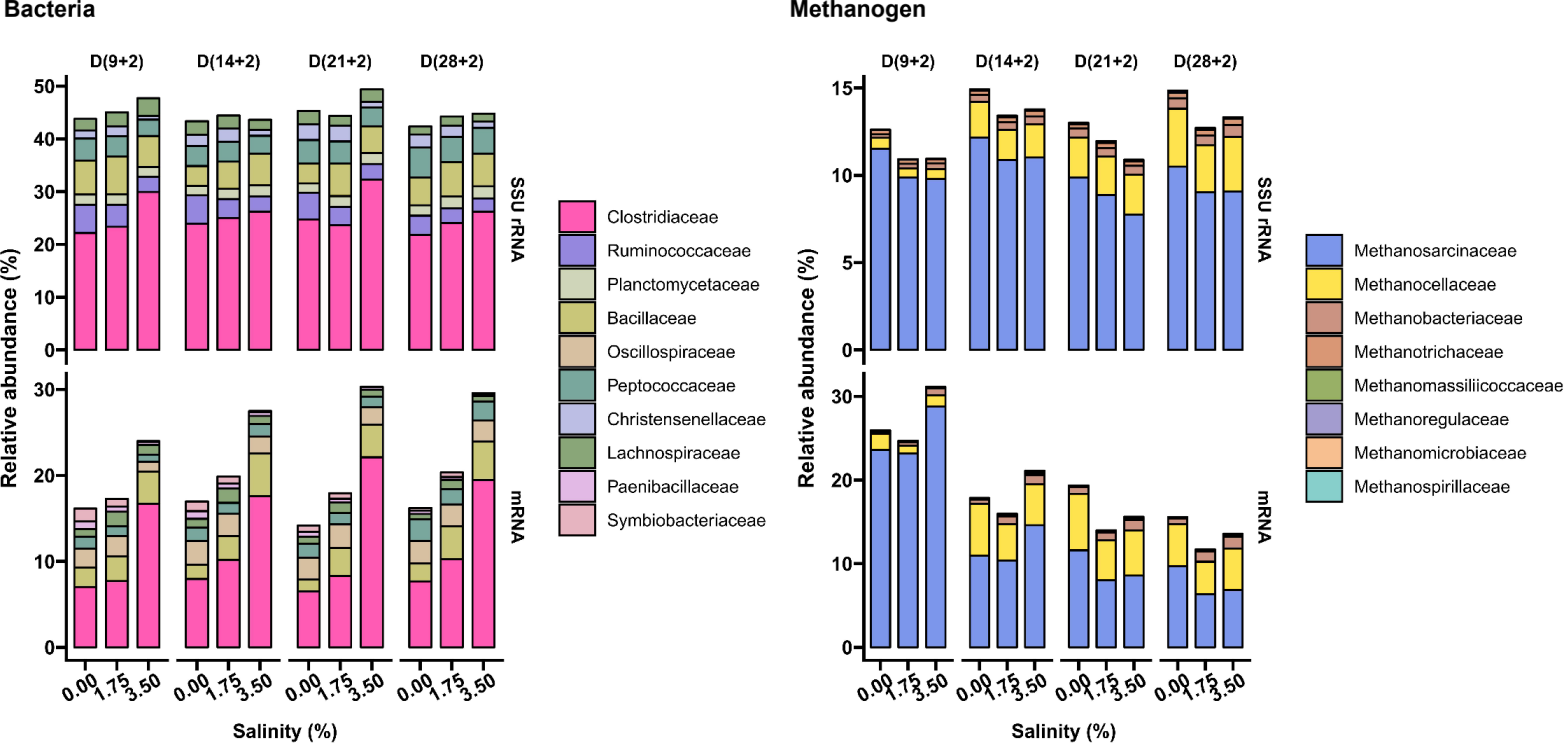
Relative abundance of particular bacterial and methanogen families based on total metatranscriptomic reads, shown across all preincubation times and salinity levels. The ten most abundant bacterial families and the eight most abundant archaeal families detectable in all samples are shown on the 16S rRNA level and mRNA levels, respectively.

Taxonomic assignments of both 16S rRNA reads and mRNA transcripts were performed to identify the composition and functional activity of microbial communities. The microbial community composition varied in response to preincubation time and salinity (Fig. 2; Tables S5 and S6). Among bacteria, *Firmicutes* emerged as the most active phylum, with relative transcript abundances ranging from 45.9% to 55.5% on the 16S rRNA level and 25.4% to 80.4% on the mRNA level (Fig. S6). In particular, the relative mRNA abundance of the *Clostridiaceae* increased at high salinity by 87.5% relative to the control, being the transcriptionally most active family-level group (Fig. 2). Among methanogens, *Methanosarcinaceae* and *Methanocellaceae* were the most abundant methanogen families on both the 16S rRNA level and the mRNA level (Fig. 2). The total transcript abundance of *Methanosarcinaceae* decreased after day (9 + 2) on the mRNA level, while that of *Methanocellaceae* increased after day (9 + 2) on both the 16S rRNA level and the mRNA level (Fig. 2).

### Salt inhibited bacterial gene expression involved in carbohydrate utilization

**Fig 3:**
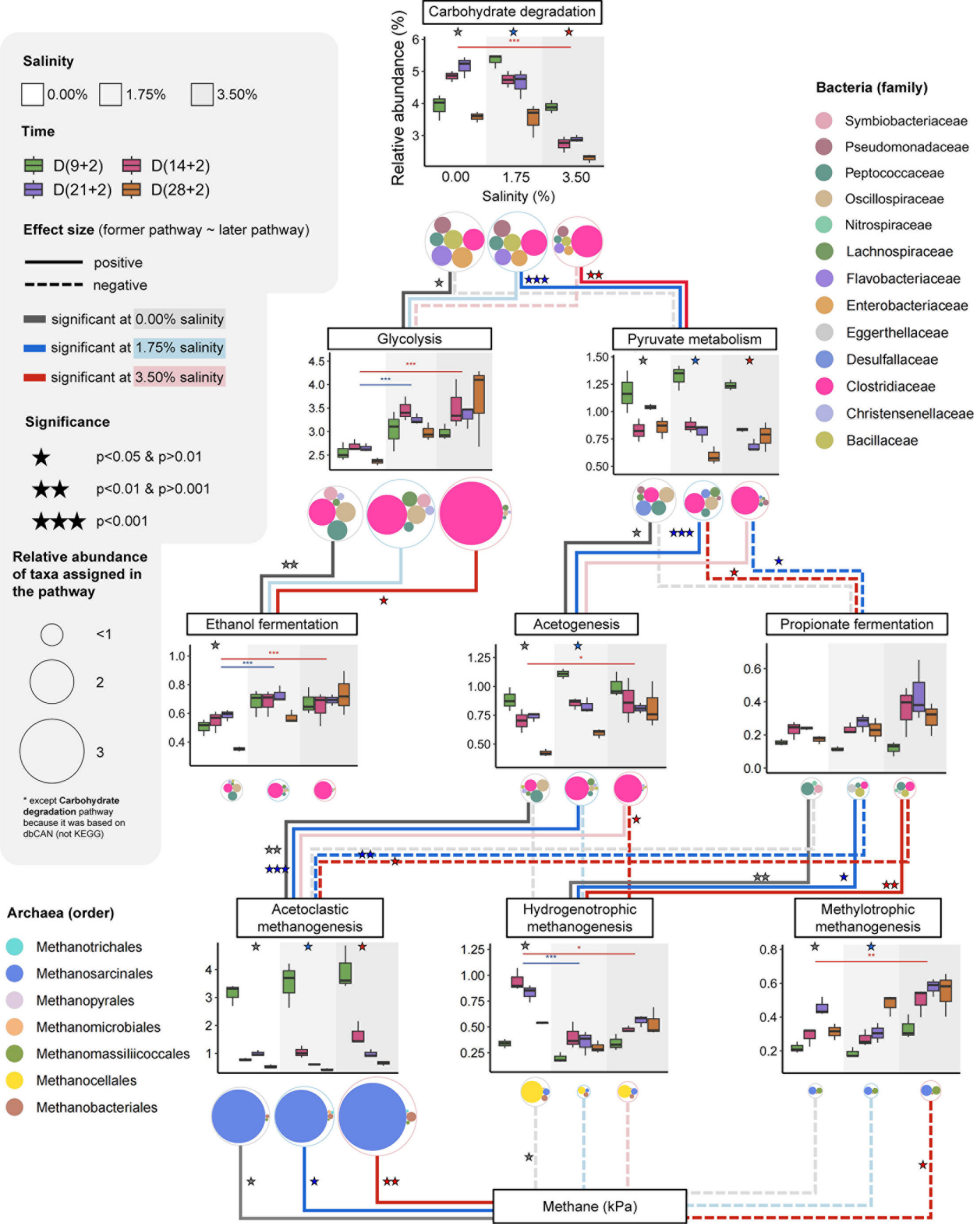
Impact of salinity on the anaerobic food web-linked functional profiles created based on triplicate metatranscriptomes for each preincubation time and salinity treatment (control [zero], 1.75%, and 3.50% salinity). Box plot headers indicate the metabolic pathways. The size of the smaller, differently colored circles beneath the box plots indicates the relative pathway-specific abundance of the transcriptionally most active families in response to increasing salinity. Taxonomic assignment of transcripts expressed by specific genes or related to particular metabolic pathways is shown in Table S7. The box plot y-axes indicate the cumulative relative transcript abundances of the metabolic pathway of interest relative to total (bacterial and archaeal) mRNA, and the x-axes indicate the different salinity levels. The four different preincubation times are color-coded. Colored stars above the box plots represent significant variations (analysis of variance [ANOVA], *P* < 0.05) in transcript abundance between the different preincubation times within the same salt treatment. Assessed by paired *t*-tests, significant differences in transcript abundance between the different salinity treatments are indicated by asterisks (*P < 0.05, **P < 0.01, ***P < 0.001). The comparison between control and moderate salinity is indicated by blue asterisks, while the comparison between control and high salinity is denoted by red asterisks. A line connecting two different metabolic pathways indicates a significant correlation between these two pathways under different salinities, i.e., the effect size of the former pathway on the latter pathway. Solid lines indicate positive correlations (effect size >0) and dashed lines indicate negative correlations (effect size <0). The color of the line indicates whether the effect is significant or non-significant, with darker lines being significant. Additionally, colored stars near the line indicate statistical significance that is associated with hypothesis tests for the regression coefficients (*n* = 12); ***P <  0.001, **P <  0.01, *P <  0.05.

We examined the mRNA transcripts of key microbial populations involved in polymer breakdown, fermentation, and methanogenesis and assessed the effect sizes along the different trophic food web levels under increasing salinity (Fig. 3; Table S7).

A total of 76,298 mRNA reads were identified to encode CAZymes, of which 49,328 reads were associated with transcripts involved in the hydrolysis of pectin, cellulose, xylan, and chitin (Table S8). The relative abundance of the CAZyme transcripts decreased under salt stress, particularly at high salinity (*P* < 0.001) (Fig. 3). In the control, the CAZyme transcript abundance peaked on day (21 + 2), but declined with increasing preincubation time and salinity (see significance values in Fig. 3). Among the carbohydrate utilization modules, the relative abundance of CAZyme transcripts for cellulose breakdown decreased under increasing salinity (*P* < 0.001), whereas the relative abundance of CAZyme transcripts other than those involved in cellulose, xylan, and chitin hydrolysis was significantly increased at both moderate salinity (*P* < 0.001) and high salinity (*P* < 0.01) (Fig. S7 and S8). The relative abundance of pectin degradation-related transcripts also significantly increased at high salinity (*P* < 0.001). Notably, *Clostridiaceae* showed significant enrichment in CAZyme expression under salt stress (*P* < 0.01) (Fig. 3; Fig. S7).

### Varied responses of fermentative populations to salt stress

Transcripts involved in polymer breakdown and pyruvate metabolism showed significant correlations under increasing salt stress (*P* < 0.01) (Fig. 3; Fig. S9). At moderate salinity, transcripts involved in central carbon (pyruvate) metabolism and decarboxylation of pyruvate to acetate were positively correlated (*P* < 0.001) (Fig. 3). The abundance of transcripts involved in pyruvate metabolism and propionate fermentation was not affected by salt stress (*P* > 0.05), but varied significantly with preincubation time (*P* < 0.05) (Fig. 3). *Clostridiaceae* became the most transcriptionally active fermentative group at the family level under salt stress, exhibiting the highest transcript levels across various metabolic pathways, including central carbon metabolism (glycolysis, pyruvate metabolism), ethanol fermentation, decarboxylation conversion of pyruvate to acetate and, to a lesser extent, propionate metabolism when exposed to high salinity (Fig. 3). Concurrently, the abundance of transcripts encoding glycolysis and ethanol fermentation increased relative to the control with high significance (*P* < 0.001).

### Salinity inhibited transcription of hydrogenotrophic methanogenesis but not methylotrophic methanogenesis

**Fig 4:**
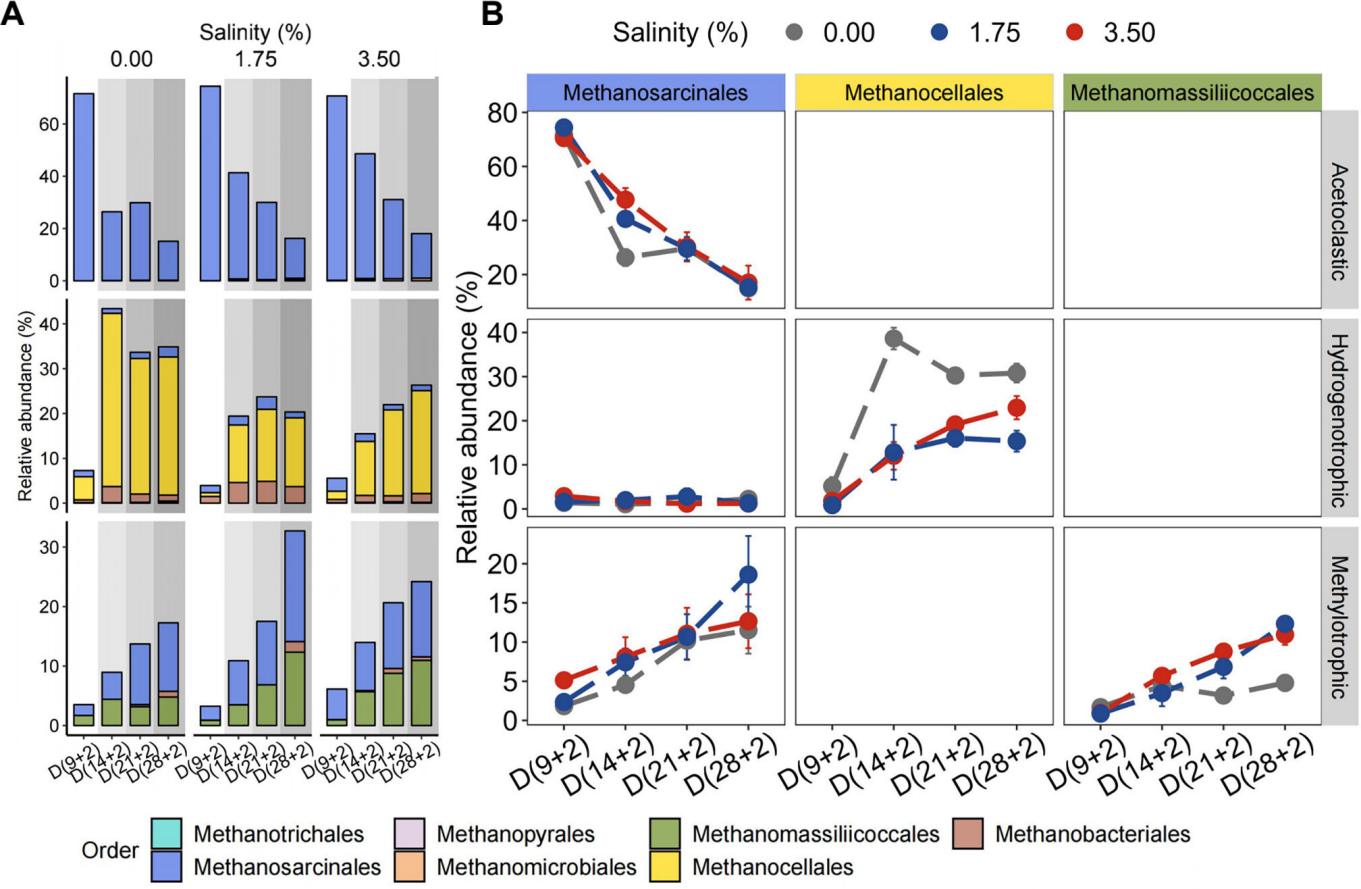
Relative gene expression of major methanogenesis pathways in response to preincubation time and salinity. (**A**) Methanogen orders that operate acetoclastic, hydrogenotrophic, and/or methylotrophic methanogenesis. The sum of total reads encoding enzymes of these three major methanogenesis pathways was set to 100% to calculate the contribution of each of these methanogen orders to a specific methanogenesis pathway across all preincubation times and salinities. (**B**) Impact of preincubation time and salinity on the order-specific abundance of transcripts involved in the acetoclastic pathway, the hydrogenotrophic pathway, and the methylotrophic pathway. Members of the *Methanosarcinales* are mixotrophic methanogens with the potential to operate all three methanogenic pathways. By contrast, *Methanocellales* spp. are specialized hydrogenotrophic methanogens, while members of the *Methanomassiliicoccales* are specialized in methylotrophic methanogenesis. Data are presented as mean  ±  s.e.m. of the estimated relative transcript abundance, *n* = 3.

Salinity inhibited transcription of hydrogenotrophic methanogenesis but enhanced methylotrophic methanogenesis. While acetoclastic methanogenesis was not inhibited, the level of its related gene expression varied significantly with preincubation time. By contrast, the expression of hydrogenotrophic methanogenesis was significantly inhibited by salinity (*P* < 0.05), particularly at moderate salinity (*P* < 0.001) (Fig. 3 and 4). The expression of the hydrogenotrophic methanogenesis pathway by *Methanocellales* peaked on day (14 + 2) in the control but was strongly suppressed by salinity at this preincubation time (*P* < 0.001) (Fig. 4B); the latter corresponding to the greatest overaccumulation of propionate relative to the control (Fig. S2). Compared to the control, a stress relief effect on the suppressed expression of the hydrogenotrophic methanogenesis pathway was evident at days (21 + 2) and (28 + 2) (Fig. 4B). Methylotrophic methanogenesis, especially via the activity of *Methanosarcinales* and *Methanomassiliicoccales,* significantly increased with preincubation time (*P* < 0.05). Specifically, the transcript abundance of the *Methanomassiliicoccales* was significantly increased relative to the control at both moderate salinity and high salinity (*P* < 0.05), defined by a significantly increased expression of the *mtaAB* gene cluster, whose transcripts are highly indicative of methanol-dependent methanogenesis. The significant increase in *mtaAB* transcripts was observed for day (28 + 2) at moderate salinity (*P* < 0.05) and across all four preincubation times at high salinity (*P* < 0.05) (Fig. S10).

### Adaptive salinity response mechanisms of key populations

The abundance of transcripts involved in osmoprotection increased under salt stress (*P <* 0.001), with higher expression at moderate salinity than high salinity (*P <* 0.001) (Fig. S11). These transcripts were primarily related to choline uptake and its conversion into betaine (Table S9). In addition, flagellin biosynthesis transcripts were significantly more abundant at high salinity than moderate salinity at days (21 + 2) and (28 + 2) (*P* < 0.001) (Fig. S12). Both osmotic stress response and flagellin synthesis were predominantly expressed by *Clostridiaceae* at high salinity (Fig. S12 and S13). The relative abundance of transcripts encoding V/A-type H^+^/Na^+^-transporting ATPase was not affected by salinity (*P* > 0.05), but shifted taxonomically from *Syntrophorhabdaceae* toward *Clostridiaceae* with preincubation time and increasing salinity (Fig. S14).

While transcripts of the methanosarcinal gene *ablB* were not detectable in the control treatments, their abundance was slightly but significantly (*P* < 0.05) increased on day (14 + 2) at moderate salinity and across all four preincubation times at high salinity (*P* < 0.05) (Fig. S15). *ablB* encodes the conversion of β-lysine into the compatible solute N^ε^-acetyl-β-lysine. Finally, *Clostridiaceae* abundance was positively correlated with the relative gene expression level of methylotrophic methanogenesis operated by *Methanosarcinales* and *Methanomassiliicoccales* (*P* < 0.01) (Fig. S16).

## DISCUSSION

To determine how salt stress affects the methanogenic food web activity and to unravel the underlying effect mechanisms, straw-amended rice field soil was taken as a model system to apply “double-RNA” metatranscriptomics after exposure of the methanogenic community to zero, moderate (1.75%), and high (3.50%) salinity for 48 hours (12, 21–23). This exposure followed a preincubation period and was designed to simulate short-term salinity shocks, such as those caused by rapid seawater intrusion events (e.g., storm surges or tidal flooding). In metatranscriptomic studies, a 48-hour exposure is considered long-term, as microorganisms typically exhibit adaptive transcriptome responses, referred to here as resilience, within this timeframe under moderate to high salt stress (25, 26). In this study, microbial resilience is defined as the capacity of microbial taxa to sustain both prevalence and functional activity under salt stress, relative to the unstressed control at each successional stage.

While the “double-RNA” approach provided comprehensive insights into the salinity effects on the methanogenic food web, the microbial communities responded more strongly on the mRNA level than on the 16S rRNA level, correlating with a faster mRNA turnover due to their shorter half-lives (12, 21–23, 27). Given the limitations of using rRNA abundance as a direct proxy for microbial activity in environmental samples (28), 16S rRNA sequencing was used solely to characterize viable community composition, while mRNA transcripts were exclusively analyzed to infer functional activity. Notably, the abundance of CAZyme transcripts was positively correlated with 16S rRNA abundance, but not with total mRNA abundance (Fig. 1D). This pattern likely reflects fundamental differences between the two molecular pools: 16S rRNA abundance is more indicative of microbial biomass and basal metabolic potential, while total mRNA encompasses a wide array of transcripts related to multiple cellular processes beyond carbohydrate metabolism (28). The observed correlation suggests that the capacity for organic matter degradation is closely linked to the abundance and activity of specific bacterial taxa capable of expressing carbohydrate-active functions under saline conditions (29). Here, we paid particular attention to sub-transcriptomes involved in polymer hydrolysis, fermentation, and methanogenesis, aiming to understand how these processes influence methane emissions under increasing salinity at different stages of community succession.

### Polymer hydrolysis and fermentation

While serving as molecular indicators of carbon acquisition strategy (30), the expression of CAZymes was significantly inhibited at high salinity (Fig. 3), accompanied by a shift toward the predominance of clostridial CAZyme transcripts across all four preincubation times (Fig. 3; Fig. S7C). The alpha diversity of CAZyme transcripts showed a significant positive correlation with CH_4_ production (*P* < 0.001) (Fig. S3 and S5), in good correspondence with the greatly varying relative expression levels of transcripts encoding specific CAZymes (Fig. S7A and S8). While the Shannon index provides a useful measure of functional diversity, it may underestimate certain aspects of functional novelty at the gene level (31). The significant increase in the relative abundance of transcripts encoding mannosidase and beta-galactosidase (*P* < 0.05) may imply a shift in the enzymatic activity from complex carbohydrate degradation toward the decomposition of simpler carbohydrates, with the latter potentially contributing to the osmotic balance or the utilization of alternative energy sources more suited to salt-stressed environments (32).

The breakdown of complex carbohydrates into monomers, followed by glycolysis to produce pyruvate, is a critical metabolic pathway for stress tolerance and signaling (33). Notably, members of the *Clostridiaceae* displayed remarkable resilience to salinity increases in native paddy soil, with *Clostridium* spp. outcompeting other fermentative bacteria across all four preincubation times under salt stress due to their metabolic capacity to gain sufficient energy for effective osmoadaptation (Tables S7 and S9). *Clostridiaceae* possess the ability to synthesize cellulosomes, multienzyme complexes for degrading plant cell wall polysaccharides, such as cellulose and hemicellulose (34, 35). In addition, *Clostridiaceae* are capable of forming endospores, a trait that facilitates survival under adverse environmental conditions, including salinity stress (36). This sporulation capacity likely contributes to their persistence in salt-affected soils (26). The reallocation of metabolic resources, reflected in the increased relative expression of genes involved in glycolysis, pyruvate metabolism, decarboxylation conversion of pyruvate to acetate, and ethanol fermentation (Fig. 2 and 3; Table S7), supports the view that *Clostridiaceae* are particularly able to adapt to salt-stressed environments.

Relative to the control, the transcription of both clostridial glycolysis and clostridial ethanol fermentation significantly increased under salt stress, with a significant link between the expression of both pathways across the four preincubation times at high salinity (Fig. 3). In glycolysis, the generation of ATP via substrate-level phosphorylation is coupled with the reduction of NAD^+^ to NADH per glucose converted into pyruvate. Ethanol fermentation regenerates NAD^+^ for glycolysis, while acetate production via acetyl-CoA from pyruvate generates additional ATP (37). The increased flow of carbon through clostridial glycolysis was most evident for day (14 + 2). Interestingly, acetate concentration was 5.6- to 5.7-fold higher under moderate and high salinity than in the control (Fig. S2C), while no inhibitory effect of increased salinity on CH_4_ production was evident (Fig. 1B). The shift toward increased acetate release by *Clostridiaceae* presumably contributed to the resilience of CH_4_ production through acetoclastic *Methanosarcinales* at day (14 + 2), in addition to the methanosarcinal ability to actively tolerate salt stress.

### Methanogenesis

The relative gene expression of the three major methanogenic pathways strongly varied across the different successional stages, depending on substrate availability and the methanogens’ ability to tolerate salt stress. Acetoclastic methanogenesis by the mixotrophic *Methanosarcinaceae* was highly active during the early stage of polymer breakdown, depending on the acetate production (15). As expected, *Methanosarcinaceae* outcompeted the obligate acetoclastic *Methanotrichaceae* during the early successional stage (Fig. 2). Interestingly, salinity did not inhibit the transcription of acetoclastic pathways in *Methanosarcinales* (Fig. 4), except during later stages (days 21 + 2 and 28 + 2), when acetate concentrations limited their acetoclastic activity (Fig. S2B). However, at day (14 + 2), the increased transcription of acetoclastic pathways under salt stress correlated with the heightened acetate concentrations (Fig. S2) and the absence of inhibitory effects on CH_4_ production (Fig. 1B). This suggests that members of *Methanosarcinales* have evolved salt tolerance mechanisms, including Na^+^ extrusion from the cytoplasm (38) and synthesis of compatible solutes such as glutamate and N^ε^-acetyl-β-lysine at moderate and high salinity, respectively (39). Indeed, the increased abundance of *ablB* transcripts at day (14 + 2) supports the notion of N^ε^-acetyl-β-lysine synthesis as a stress resilience mechanism (Fig. S15). This is also in good correspondence to the high stress resilience of *Methanosarcinales* at that preincubation time (Fig. 1A and 4). *Methanosarcina* spp. are able to uptake the widely distributed osmoprotectant “glycine betaine” (40), which rapidly conveys strong osmoprotection to their acetoclastic activity (41).

Hydrogenotrophic methanogens, particularly *Methanocella* spp., are intrinsically adaptive to low H_2_ concentrations (42) and are frequently involved in the syntrophic oxidation of propionate in rice paddies (12, 14). Under salt stress, the transcription of mRNA encoding hydrogenotrophic methanogenesis was strongly repressed. However, relative to the other methanogenesis pathways, the inhibitory effect on the expression of hydrogenotrophic methanogenesis decreased with preincubation time; most evident particularly at high salinity (Fig. 4). The greatest inhibitory effect occurred at day (14 + 2), at which the net consumption of propionate by *Methanocellaceae-*driven syntrophic methanogenesis starts to occur under no-salt conditions (12, 14). Correspondingly, propionate accumulated to significantly higher concentrations under salt stress than in the control treatment (Fig. S2B). Thus, the fact of no salinity-induced inhibition of CH_4_ production on day (14 + 2) must be due to highly active acetoclastic methanogenesis (Fig. 1B), a view well supported by the significantly increased relative expression of the acetoclastic methanogenesis pathway at that preincubation time (Fig. 4B). Specifically, the salt tolerance of cultured *Methanocella* spp. has been shown not to exceed 20 g/L NaCl in the medium (43, 44). Correspondingly, *Methanocellaceae* was shown to withstand 2% salinity in environmental settings, but not 3% salinity (45, 46). Given these previous findings, it may be surprising that, relative to the control, the salinity-affected transcription of mRNA encoding *Methanocellaceae*-driven methanogenesis was less suppressed at days (21 + 2) and (28 + 2) than day (14 + 2) (Fig. 4), with CH_4_ production during the 2-day salinity exposure of no less than 50% of the control treatment (Fig. 1B). In addition to the negative impact of salinity on the transcriptional activity of the *Methanocellaceae*, salt stress may have affected the actual production of propionate and the activity of the limited number of bacterial species capable of syntrophic propionate oxidation, such as *Syntrophobacterium* spp*.* and *Pelotomaculum* spp*.* (11).

In contrast, the expression of methylotrophic methanogenesis steadily increased with preincubation time. Intriguingly, this increase in transcript abundance was related to both H_2_-independent methylotrophy by *Methanosarcinaceae* and H_2_-dependent methylotrophy by *Methanomassiliicoccales* (47, 48). The increased transcription of mRNA encoding methanosarcinal methylotrophy may be explained by the above-discussed ability of *Methanosarcina* spp. to effectively cope with salt stress. In contrast to *Methanosarcinaceae*, the transcription of mRNA encoding methylotrophy by *Methanomassiliicoccales* was significantly increased relative to the control. Comparative analysis of metagenome-assembled genomes has shown that members of the *Methanomassiliicoccales* possess the potential to synthesize trehalose and therefore are able to cope with increased salinity (49). As evidenced by the significant abundance increase in *mtaAB* transcripts, particularly at high salinity (Fig. S10), methanol plays a major role in the methanogenic community response to increased salinity. Under anaerobic conditions, certain amounts of methanol will become available through the dimethoxylation of pectin, while another potential methanol source may be lignin decomposition (50, 51).

### A global view on the salinity-impacted methanogenic food web in paddy soil

Various studies have reported an inhibitory effect of increased salinity on CH_4_ production (6, 52–54), but none have shown that the salinity effect on the food web-related gene expression depends on the successional stage. Moreover, many studies investigating salinity effects on methanogenesis are confounded by the presence of sulfate, which is prevalent in SWISLR events (55, 56). In this study, we focused specifically on the impact of NaCl by maintaining sulfate-free conditions, allowing for a clearer understanding of how salinity alone affects microbial metabolism within the methanogenic food web. Methanogenic communities strategically allocate resources to pathways that prioritize energy generation, redox balance, and osmotic stress response, potentially by regulating carbon flux to specific fermentative pathways (57). In our study, this metabolic reprogramming was associated with a highly significant increase in the transcriptional activity of the *Clostridiaceae*, which directly contributes to the resilience of acetoclastic CH_4_ production by *Methanosarcina* spp. under salt stress. Intriguingly, methanol-dependent methanogenesis through both *Methanosarcinaceae* and *Methanomassiliicoccales* seemed to be favored under salt stress, as evidenced by the significantly increased transcript abundance of the *mtaAB* genes. The salinity-induced metatranscriptomic increase in the transcriptional activity of *Clostridiaceae* and *Methanomassiliicoccales* may be functionally linked at high salinity (Fig. S16). Members of the *Methanomassiliicoccales* have the genetic potential to assimilate acetate and ethanol as carbon sources for growth (49), which corresponds well to the salinity-induced relative increase in the clostridial transcription of mRNA related to glycolysis, decarboxylation of pyruvate to acetate, and ethanol fermentation. Moreover, recent research has shown that extracellular electron transfer can facilitate CH_4_ production in a co-culture of *Clostridium* and *Methanomassiliicoccus* strains (58), underscoring the complex interplay between fermentation and methanogenesis in salinity-affected environments. Nonetheless, we acknowledge that food web structures inferred from these data remain putative, and future studies incorporating stable isotope probing or single-cell approaches are needed to trace carbon fluxes and confirm interspecies metabolite transfers.

### Conclusion

Our study presents novel knowledge of the impact of salt stress on the viable community and their activity within the methanogenic food web, leading to the inhibition of CH_4_ and CO_2_ production; specifically, that key microbial populations exhibit dynamic metabolic responses depending on community successional stage and salinity level. By employing a “double-RNA” metatranscriptomic approach, we captured real-time shifts in carbon flux within this food web, showing how functional guilds change in response to salt stress during community succession (12, 21–23). We identified critical linkages between salt-sensitive populations and metabolic pathways that directly influence CH_4_ production, with a particular emphasis on how *Clostridiaceae* and *Methanosarcina* spp. mediate stress resilience of their metabolic activities. The identified functional links between predominant populations, which effectively cope with salt stress, offer valuable insights for future studies on methanogenesis under environmental changes, particularly in understanding whether effects occur solely under salt stress or in response to other stressors.

## MATERIALS AND METHODS

### Soil microcosms

Soils were collected from drained rice fields at the Italian Rice Research Institute (IRRI) as described previously (14). The IRRI is located in the valley of the river Po near Vercelli (Italy). To ensure standardized sampling without seasonal crop effects, sampling was conducted after the rice growing season. The surface soils (top 10 cm) were collected using a random sampling approach at multiple field locations to ensure representative sampling. The physicochemical characteristics of the Italian paddy soils have been previously described (59). After collection, soils were air-dried, mechanically crushed, and sieved to <2 mm to remove debris and stones and stored in sealed plastic bags at room temperature until the experiment was set up. The drying process was done carefully to prevent any changes in microbial communities while allowing for long-term storage prior to the experiment.

Slurry microcosms were set up by filling 40 g of homogenized dry soil with 35 mL of autoclaved water (maintaining an 8:7 soil:water ratio) and 0.5 g rice straw (1–2 cm) in 125 mL autoclaved bottles (14). The rice straw was added as the main carbon source with a carbon content of 30%–40% and a nitrogen content of 0.6%–0.7%. The thoroughly mixed slurries were sealed with butyl rubber stoppers and repeatedly flushed with N_2_ to establish anoxic conditions. Prior to the salinity treatments, slurries were preincubated for 9, 14, 21, and 28 days in the dark at 30°C, respectively (Fig. S1). Sodium chloride was dissolved in 5 mL autoclaved water to adjust slurries to a concentration of 1.75% and 3.50%, corresponding to half and full seawater salinity, and falling within the reported range of global average seawater intrusion (60–62). A total of 12 treatments were established, with three replicates per treatment, resulting in 36 bottles in total. A gas sample (0.1 mL) was taken immediately after salt addition and vigorous shaking of the slurries. Then the salty slurries were incubated for 2 days in the dark without shaking. To isolate the effects of salinity on the methanogenic food web, salinity was adjusted solely with NaCl, while MgSO_4_ was excluded to prevent activation of sulfate-reducing bacteria, following a recent study on the osmoregulation of freshwater anaerobic methane-oxidizing archaea under salt stress (63). After the 48-hour salt treatment, slurry material beneath the topsoil layer was collected from all microcosms, with the residual rice straw being withdrawn. The slurry samples (day [9 + 2], day [14 + 2], day [21 + 2], day [28 + 2]) were immediately shock-frozen using liquid N_2_ and then stored for molecular analysis at –80°C. In addition, gas samples (0.1 mL) and liquid samples (0.5 mL) were taken from the same set of slurries for metabolite measurements. All 36 microcosms (3 replicate microcosms × 4 time points × 3 salt treatments) were destructively sampled after the salt stress treatments. Independent analysis of each set of triplicate slurries included process measurements and metatranscriptomic analysis using the double-RNA approach.

### Metabolite measurements

A GC-8A gas chromatograph (Shimadzu, Duisburg, Germany) containing a Haysep Q column was used to measure CH_4_ and CO_2_. Data were analyzed with PeakSimple software (SRI Instruments, Bad Honnef, Germany) and calculated by linear regression as described previously (14). Concentrations of acetate and propionate in the liquid sample of the soil slurries were measured by high-performance liquid chromatography equipped with an ion-exclusion column (Aminex HPX-87-H, BioRad, München, Germany) and coupled to a UV–Vis detector (Sykam, Fuerstenfeldbruck, Germany) (64).

### “Double-RNA” metatranscriptomics

Total RNA was manually extracted from slurries using a previously established method (12, 14). cDNA libraries were generated from the RNA extracts without prior removal of rRNA, and subsequently sequenced using the Illumina HiSeq platform. Detailed protocols for RNA extraction and library preparation are provided in the Supplemental methods.

The total RNA reads were analyzed using a customized metatranscriptomic pipeline (see Supplemental material) (12, 14). Following raw data cleaning, 16S rRNA reads were extracted using SortMeRNA 2.0 (65) with SILVA (release 138). Taxonomic analysis was performed using two approaches: 16S rRNA reads were processed with QIIME 2 (66), while mRNA reads were taxonomically assigned using the NCBI non-redundant protein database by MEGAN6 (67). Functional annotation and further sub-annotation were exclusively based on mRNA reads using the Kyoto Encyclopedia of Genes and Genomes (KEGG) and SEED databases (67–69).

The annotation of CAZyme encoding transcripts was achieved against dbCAN2 (70) using DIAMOND (71) following previous protocols (14). Briefly, functional CAZyme modules (e.g., cellulose, chitin, xylan, pectin, and other hemicellulose degradation) were defined by grouping enzymatic functions based on their enzyme commission numbers. To facilitate annotation, a comprehensive mapping file was generated from all available dbCAN entries, formatted as an indexed SQLite database, and queried using custom Python scripts. mRNA reads were first aligned to dbCAN to identify top hits, which were then matched against the curated mapping file to assign them to specific CAZyme modules. In-depth sub-transcriptome analysis and taxonomic reassignment were conducted using in-house scripts as previously described (17).

### Statistical analyses

Non-metric multidimensional scaling (NMDS) plots were generated in the R (version 4.3.1) software (R Development Core Team, 2021) using the vegan package (version 2.6.2) (70) with the metaMDS function and Bray–Curtis dissimilarity matrices based on relative transcript abundance. We conducted a permutational analysis of variance using the adonis2 function from the vegan package to test whether community differences on both 16S rRNA and mRNA levels between (i) the four incubation time points, (ii) the three different salinity treatments, and (iii) their interaction, in the NMDS ordinations are statistically significant (70). The linear discriminant analysis effect size was used to estimate microbial indicators separately for each time point and each salt treatment, defined as significantly enriched (72). To assess temporal variation in transcript abundance within each salinity treatment (Fig. 3), one-way analysis of variance (ANOVA) was performed independently for each salinity level across the four preincubation time points. Prior to conducting ANOVA, assumptions of normality and homogeneity of variances were tested using the Shapiro-Wilk and Levene’s tests, respectively. In cases where these assumptions were violated, the non-parametric Kruskal-Wallis test was employed. To investigate functional responses to salinity (Fig. S11), differential expression analysis was performed using the DESeq2 package (v1.40.2) based on raw read counts annotated with KEGG and SEED level 3 functional categories (73). Wald tests were used to determine statistical significance, and resulting *P*-values were adjusted using the Benjamini-Hochberg false discovery rate method (74). This analysis was specifically applied to generate the functional profiles as shown in Fig. S11.

### Treatment effects analyzed by linear model

We employed linear models to separately evaluate the impact of each salinity treatment, considering the complete independence of the variables (preincubation time and salt treatment). Each coefficient reflects the effect size of the associated predictor variable, and the *P* value associated with each coefficient tests the null hypothesis that the corresponding coefficient is equal to zero (no effect). A *P* value <0.05 suggests that the coefficient is statistically significant and has an impact.

## Data Availability

The raw sequence data reported in this study are openly available in the NCBI Sequence Read Archive (BioProject PRJNA415619).

## SUPPLEMENTAL MATERIAL

Supplemental Material msystems.00017-25-s0001.pdf Figures S1 to S16, Tables S1 to S9, and supplemental methods.

ASM does not own the copyrights to Supplemental Material that may be linked to, or accessed through, an article. The authors have granted ASM a non-exclusive, world-wide license to publish the Supplemental Material files. Please contact the corresponding author directly for reuse.
