# Supplementary material for: Time-series metatranscriptomics reveals differential salinity effects on the methanogenic food web in paddy soil
Source: mSystems. 2025 Jul 21;10(8):e00017-25. doi: 10.1128/msystems.00017-25 (PMC12363242; doi:10.1128/msystems.00017-25)
Supplement: Supplemental Material — Figures S1 to S16, Tables S1 to S9, and supplemental methods. [file msystems.00017-25-s0001.pdf]

Supplemental material

Time-series metatranscriptomics reveals differential salinity effects on the methanogenic food web in paddy soil

Xi Zhou[a,b], Xin Li[c], Qicheng Bei[d], Xingjie Wu[a], Guihua Xu[e], Xiuzhu Dong[f], Werner Liesack[g], Zhenling Cui[a], Fusuo Zhang[a], Jingjing Peng[a*]

Supplemental Figures S1 to S16 **pages 2 - 22**

Supplemental Tables S1 to S9 **pages 23 - 58**

Supplementary Method **pages 59 – 63**

[a] State Key Laboratory of Nutrient Use and Management, College of Resources and Environmental Sciences, National Academy of Agriculture Green Development, China Agricultural University, 100193, Beijing, China.

[b] State Key Laboratory of Efficient Utilization of Arid and Semi-arid Arable Land in Northern China, Beijing, 100081, China.

[c] Institute of Agricultural and Nutritional Sciences, Martin-Luther-Universität Halle-Wittenberg, Betty-Heimann-Strasse 5, Halle (Saale), 06120, Germany.

[d] Department of Biological Sciences, University of Southern California, Los Angeles, CA, 90089-0371, USA.

[e] School of Light Industry Science and Engineering, Beijing Technology and Business University, Beijing, 100048, China.

[f] State Key Laboratory of Microbial Resources, Institute of Microbiology, Chinese Academy of Sciences, 100101, Beijing, China

[g] Max Planck Institute for Terrestrial Microbiology, Marburg, 35043, Germany.

*Address correspondence to jingjing.peng@cau.edu.cn (J.J. Peng)

Supplemental Figures

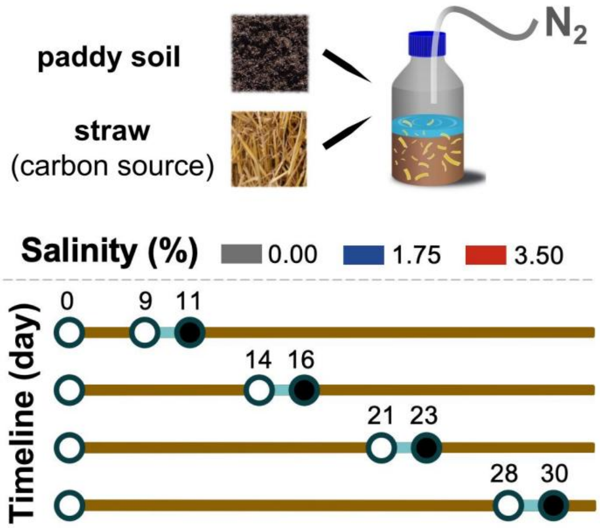

Figure S1 Detailed information on the experimental design. The experimental setup involved slurry microcosms incubated under anoxic conditions, with the addition of rice straw as a carbon source. Salt stress was set at control (0.00%), moderate (1.75%) and high (3.50%) salinity. The pre-incubation period without salt stress was for 7, 14, 21, and 28 days, followed by stress exposure for 2 days. Blue lines represent the salinity exposure period. Solid black circles indicate the time point at which the slurry microcosms were sampled destructively for molecular analysis and metabolite measurements.

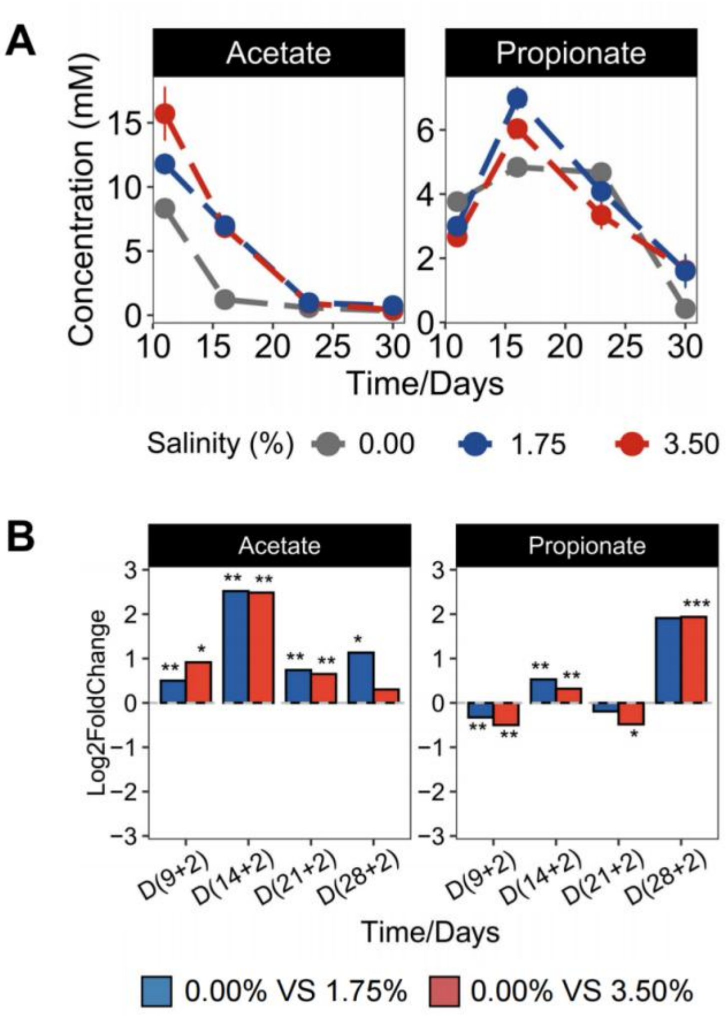

Figure S2 Concentrations of acetate and propionate in response to salt stress after the four different preincubation time points. (A) Absolute concentrations of acetate and propionate at the three salinity treatments (0.00%, 1.75%, 3.50%) (means ± SE, n = 3). Please note that the dashed lines connecting the preincubation times are only shown for improved visualization of the overall results. (B) Bar plots display the effects of salt stress on acetate and propionate as $\log_2$ fold change relative to the control treatment.

Statistical significance of difference was assessed using the paired t-test. Positive values indicate a salinity-induced enrichment relative to the the control. Significance is denoted by asterisks (* $P < 0.05$, ** $P < 0.01$, *** $P < 0.001$); non-significant comparisons (ns) are not shown for clarity.

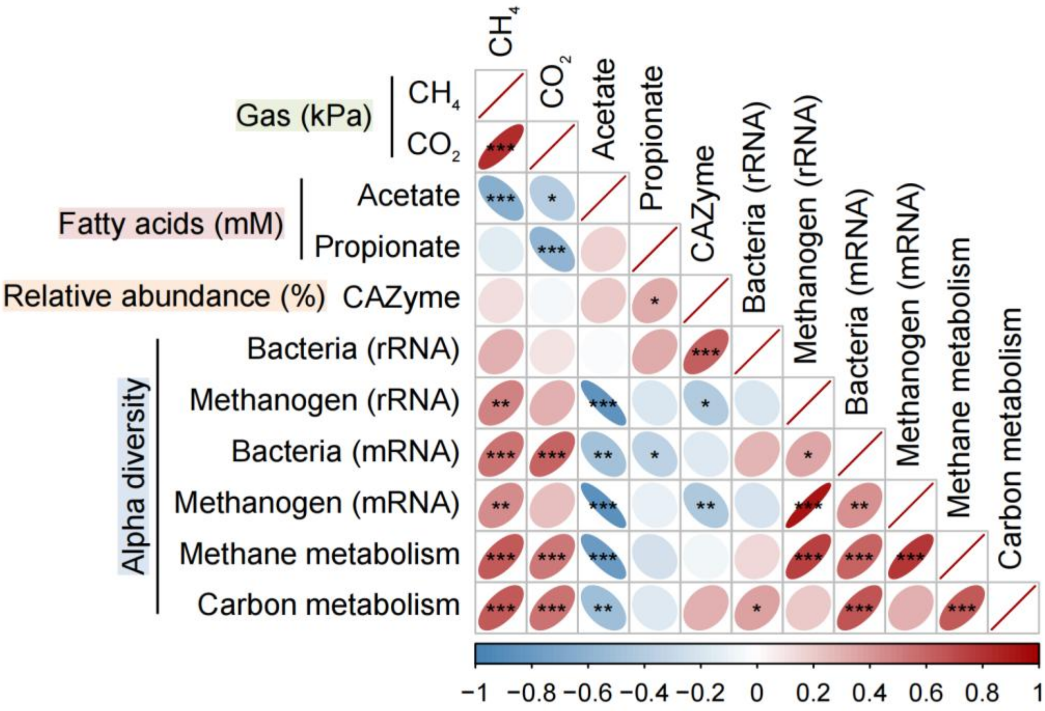

Figure S3 Correlation matrix of the relationships between environmental parameters (CH_4, CO_2, acetate, propionate), microbial communities (relative CAZyme transcript abundance), and the alpha diversity (Shannon index) of the following variables analyzed across all three treatments: bacterial and methanogen rRNA, bacterial and methanogen mRNA, as well as KEGG methane and carbon metabolisms. Notable correlations are highlighted with color intensity indicating strength and direction (positive, red; negative, blue). Asterisks indicate significant correlations with * $P < 0.05$, ** $P < 0.01$, and *** $P < 0.001$; non-significant comparisons (ns) are not shown for clarity.

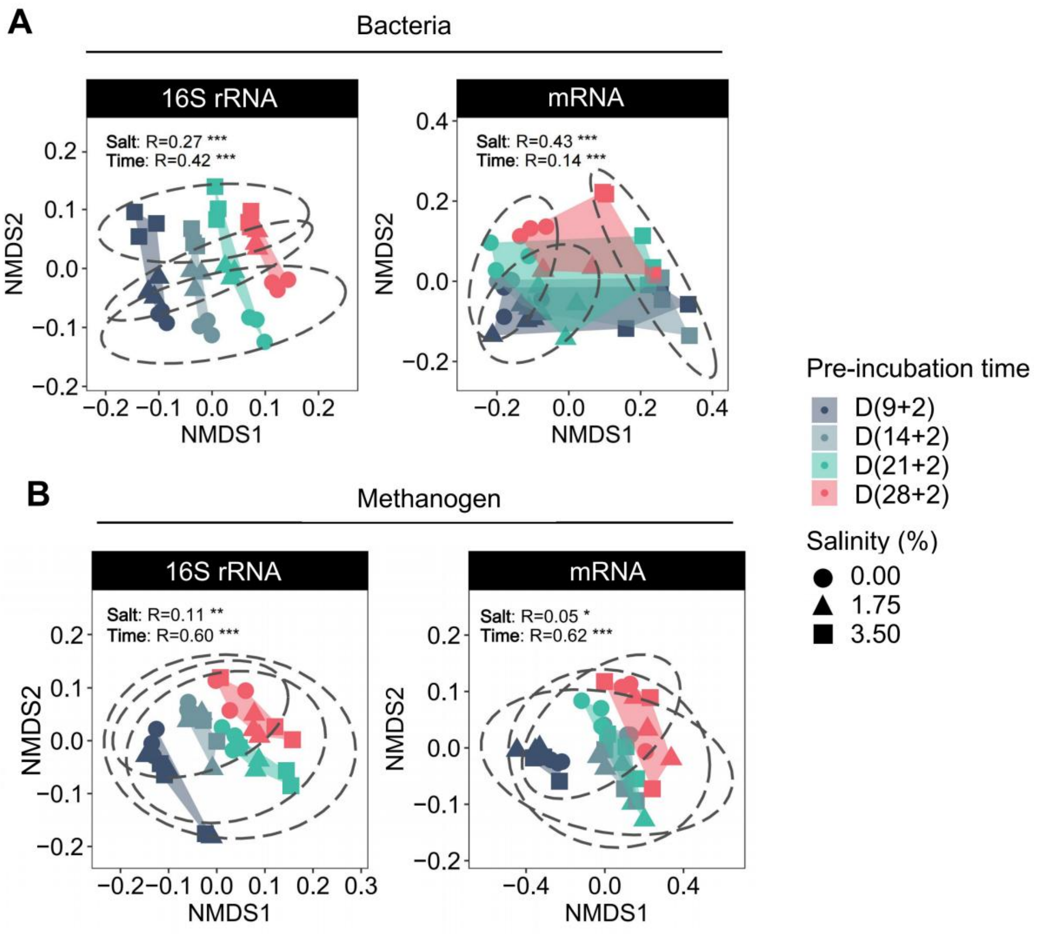

Figure S4 NMDS analysis based on Bray-Curtis similarity matrices separately shown for (A) the bacterial community and (B) the methanogens at 16S rRNA and mRNA levels. The analysis is based on non-metric multidimensional scaling (NMDS) using weighted UniFrac distances calculated separately for the bacterial community and the methanogens (stress < 0.2). The colors indicate the community distribution in response to the four different preincubation times, while the shape of the symbols and the dashed ellipses

show the commuity distribution in response to the different salinities. The significance values are based on permutational analysis of variance (PERMANOVA) for salinity exposure and preincubation time. Significance is denoted by asterisks (* $P < 0.05$, ** $P < 0.01$, *** $P < 0.001$).

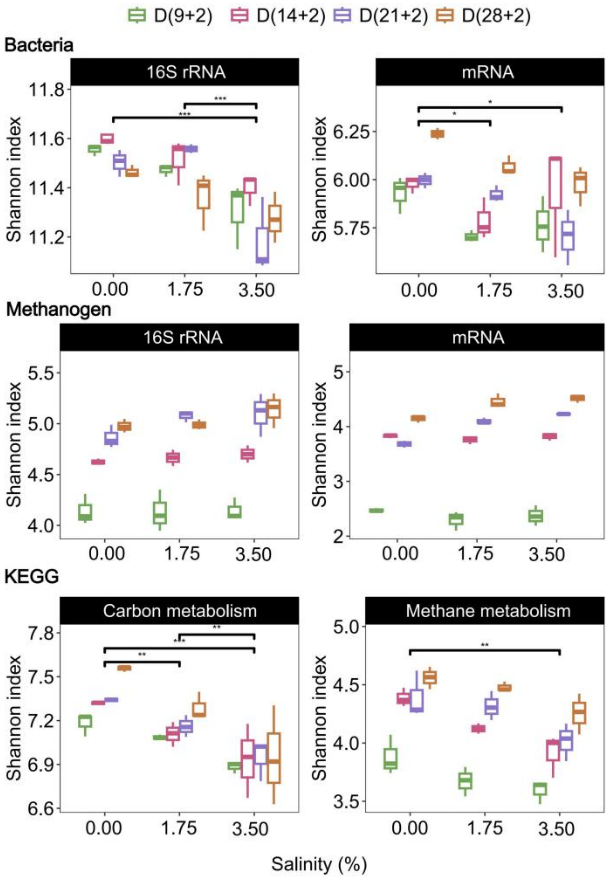

Figure S5 Alpha diversity of metatranscriptomic 16S rRNA and mRNA shown separately for the (A) bacterial community and (B) methanogens, as well as for (C) carbohydrate metabolism and methane metabolism based on KEGG. The y axes indicate the Shannon index and the x axes indicate the different salinities. The four preincubation times are indicated by different colors. Statistical significance of difference was assessed using the paired t-test. Asterisks indicate significant difference of the Shannon index between the treatments (* $P < 0.05$, ** $P < 0.01$, *** $P < 0.001$); non-significant comparisons (ns) are not shown for clarity.

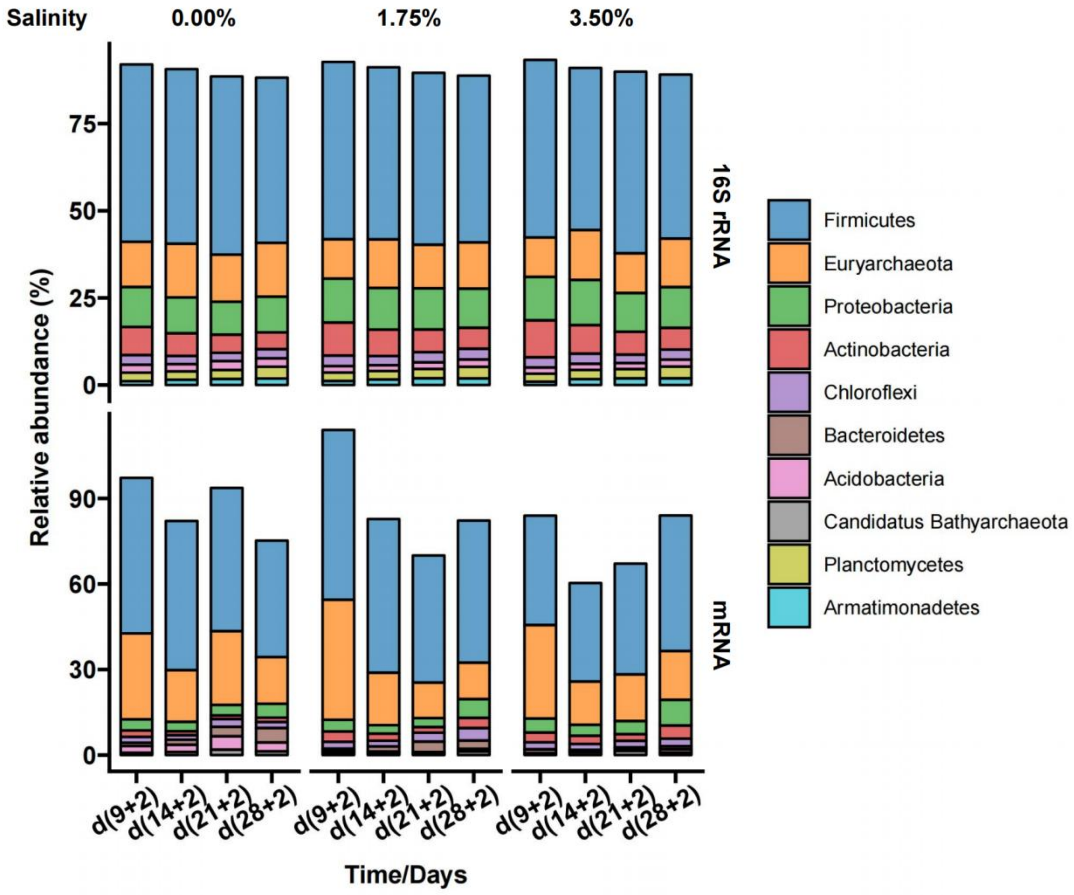

Figure S6 Taxonomic assignment of total metatranscriptomic 16S rRNA and mRNA (top 10 taxa).

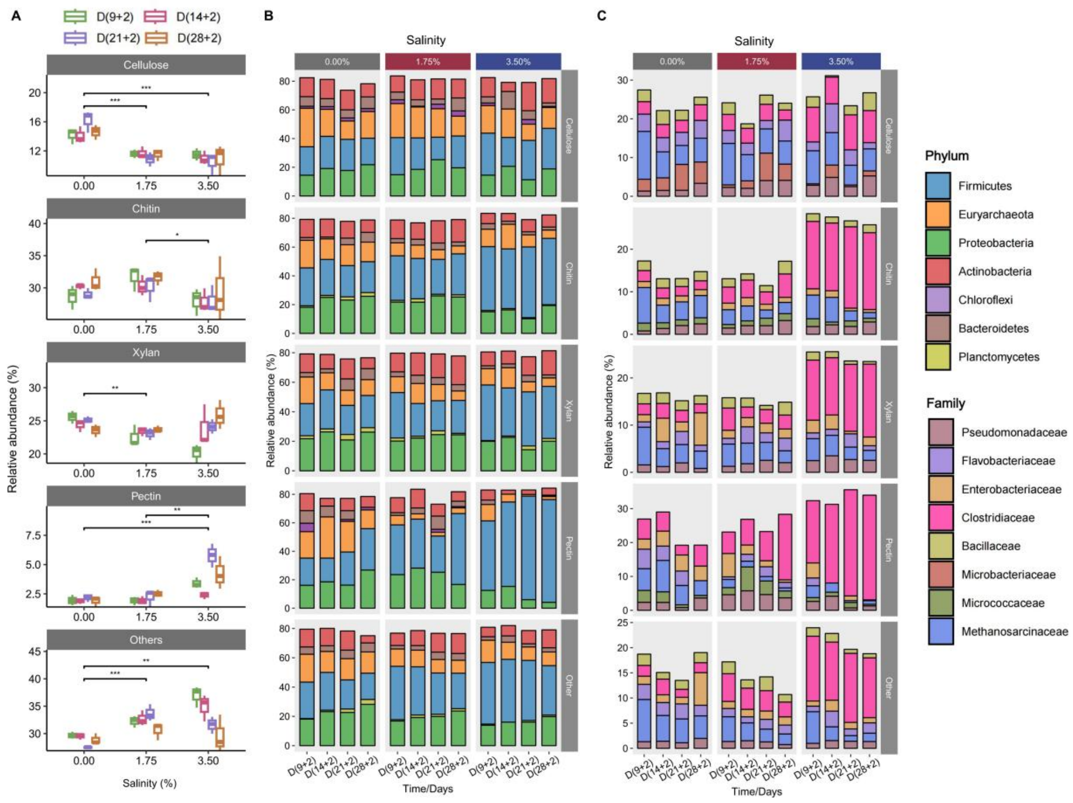

Figure S7 Relative transcript abundance of functional CAZyme modules and their taxonomic composition at the phylum and family levels in response to the four preincubation times and the three salinity treatments. (A) Relative transcript anundance of CAZyme modules functionally categorized into those specific for the decomposition of cellulose, chitin, xylan, pectin, and others. The relative transcript abundance is calculated in relation to total CAZyme transcripts set as 100%. Asterisks indicate significant difference between treatments (* $P < 0.05$, ** $P < 0.01$, *** $P < 0.001$); non-significant comparisons (ns) are not shown for clarity. (B, C) Taxonomic composition of the five functionally categorized CAZyme modules at (B) the phylum level and (C) the

family level.

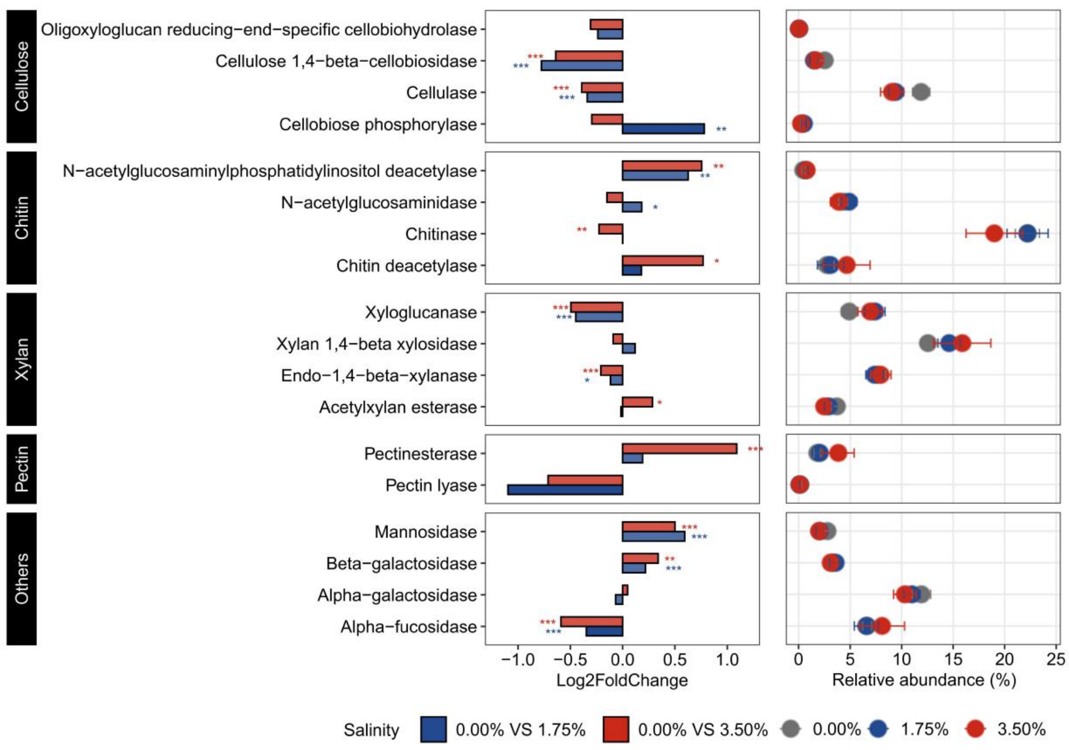

Figure S8 Salinity-induced changes in relative transcript abundance of specific CAZymes. The specific CAZyme functions were grouped according to the target polymer (cellulose, chitin, xylan, pectin, others). Their changes in relative transcript abundance are shown as $\log_2$ fold change relative to the control treatment. The paired t-test was used to test for statistical significance of difference. Significance of salinity-induced changes in relative transcript abundance is denoted by asterisks (* $P < 0.05$, ** $P < 0.01$, *** $P < 0.001$); non-significant comparisons (ns) are not shown for clarity.

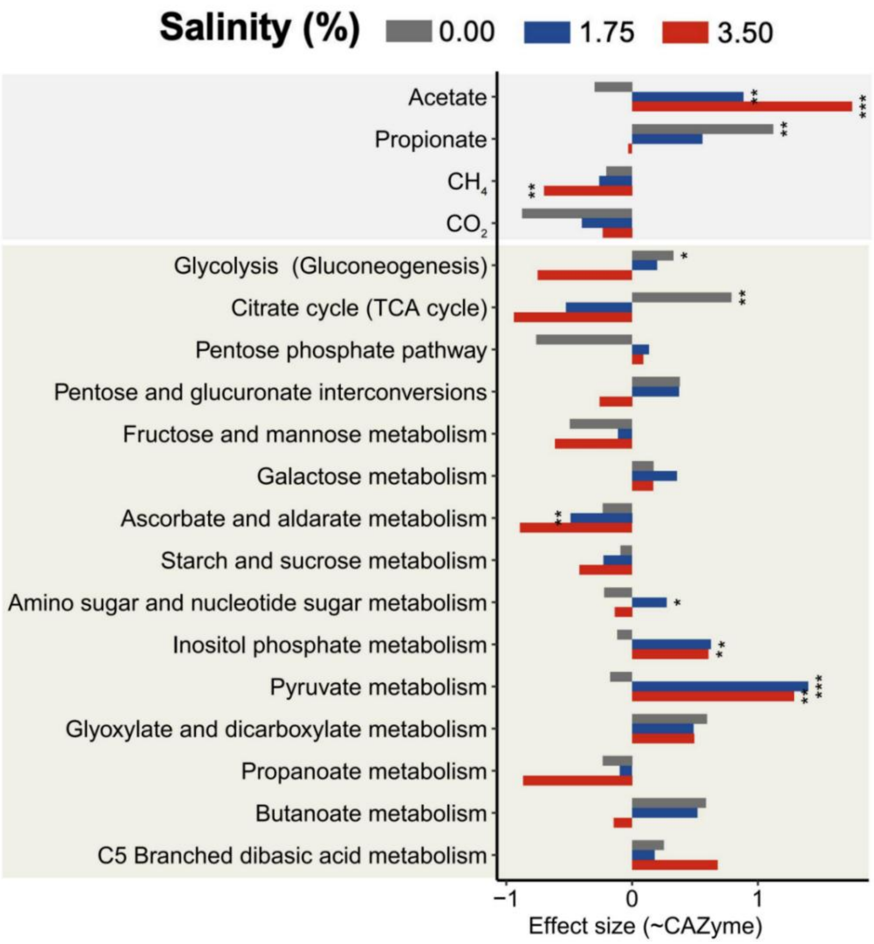

Figure S9 Effect sizes of CAZyme transcript abundance on environmental parameters (CH_4, CO_2, acetate, propionate) and the relative abundance of transcripts encoding cellular functions involved in carbohydrate metabolism. The effect sizes were calculated for the different salinity treatments as indicated by different colors. Data are presented as mean of the estimated effect sizes (n = 12). Statistical significance is based on Wald type II χ^2 tests: * $P < 0.05$. ** $P < 0.01$, *** $P < 0.001$; non-significant comparisons (ns) are not shown for clarity.

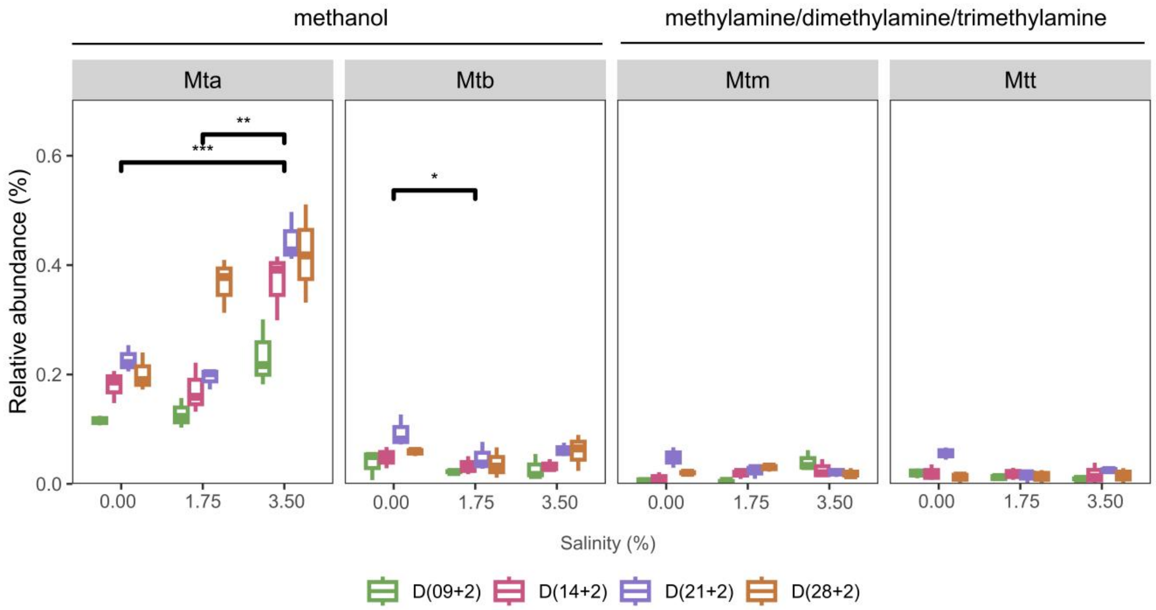

Figure S10 Differential abundance analysis of transcripts encoding methylotrophic methanogenesis, including methanol-dependent (*mtA*) and methylamine/dimethylamine/trimethylamine-dependent (*mtb*, *mtm*, and *mtt*) methanogenesis. The relative abundances are shown in relation to total metatranscriptmic mRNA. Statistical significance of difference was assessed using the paired t-test. Asterisks indicate significant difference between the treatments (* $P < 0.05$, ** $P < 0.01$, *** $P < 0.001$); non-significant comparisons (ns) are not shown for clarity.

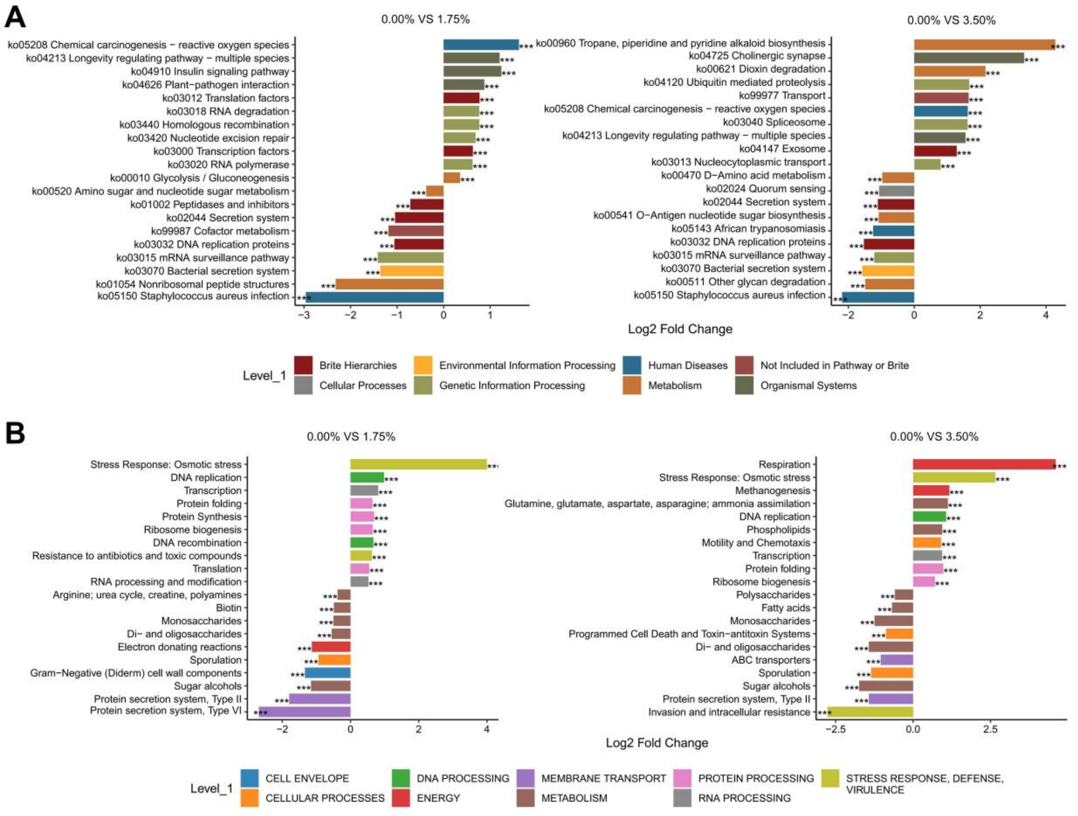

Figure S11 Differential abundance analysis of transcripts affiliated to (A) KEGG and (B) SEED databases at particular level 3 categories. The bar plots display the salinity-induced changes in transcript abundance as log2fold changes relative to the control treatment. Positive values indicate a salinity-induced relative metatranscriptomic enrichment of transcripts. Asterisks indicate statistically significant differences between treatments based on Wald tests performed in DESeq2 (* $P < 0.05$, ** $P < 0.01$, *** $P < 0.001$). All the resulting P values were adjusted for multiple testing using the Benjamini-Hochberg false discovery rate method.

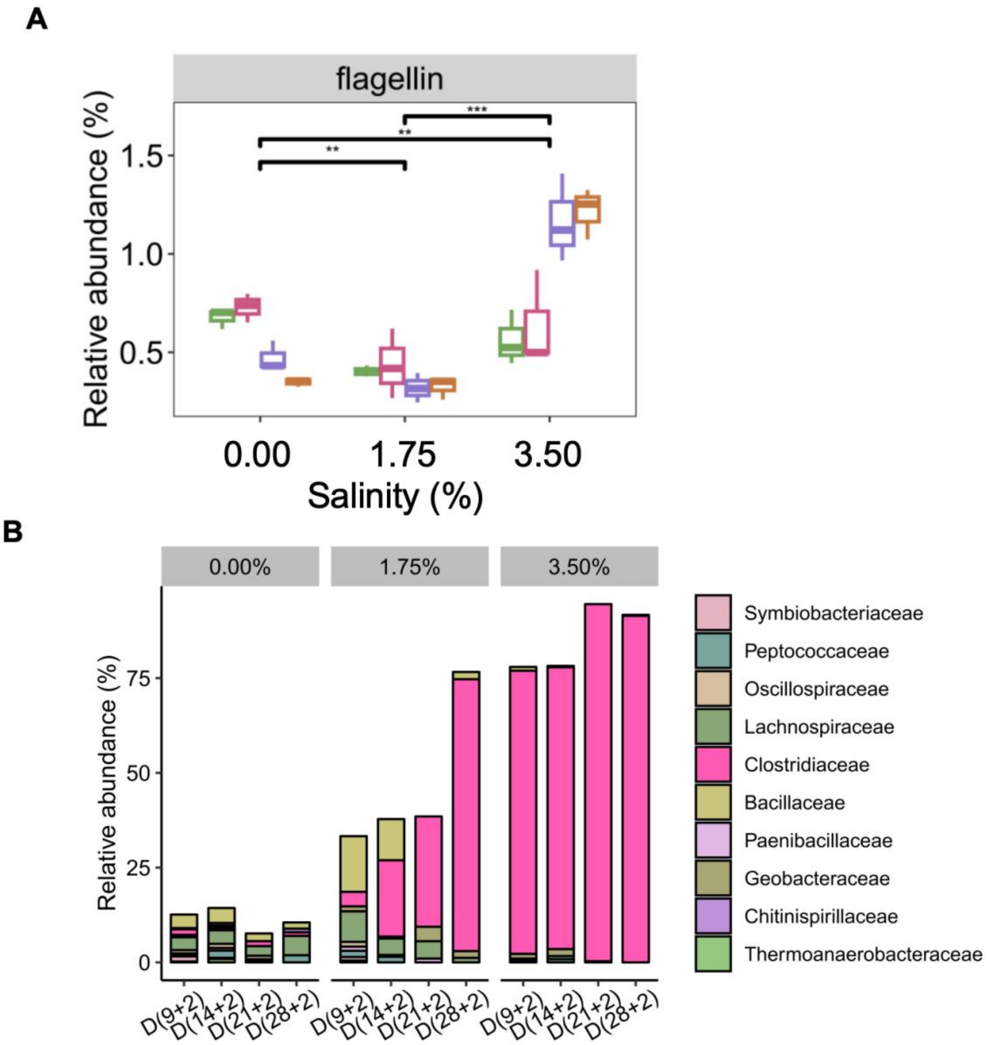

Figure S12 Differential abundance analysis of flagellin-encoding transcripts and their taxonomic affiliation. The KEGG IDs and the exact gene names are listed in Table S9. (A) Relative abundance of flagellin-encoding transcripts in response to preincubation time and salinity treatments, shown in relation to total metatranscriptomic mRNA. Asterisks indicate significant difference between the treatments (* $P < 0.05$, ** $P < 0.01$, *** $P <$

0.001). (B) Taxonomic composition of the flagellin-encoding transcripts at the family level (top 10) in response to preincubation time and salinity treatments.

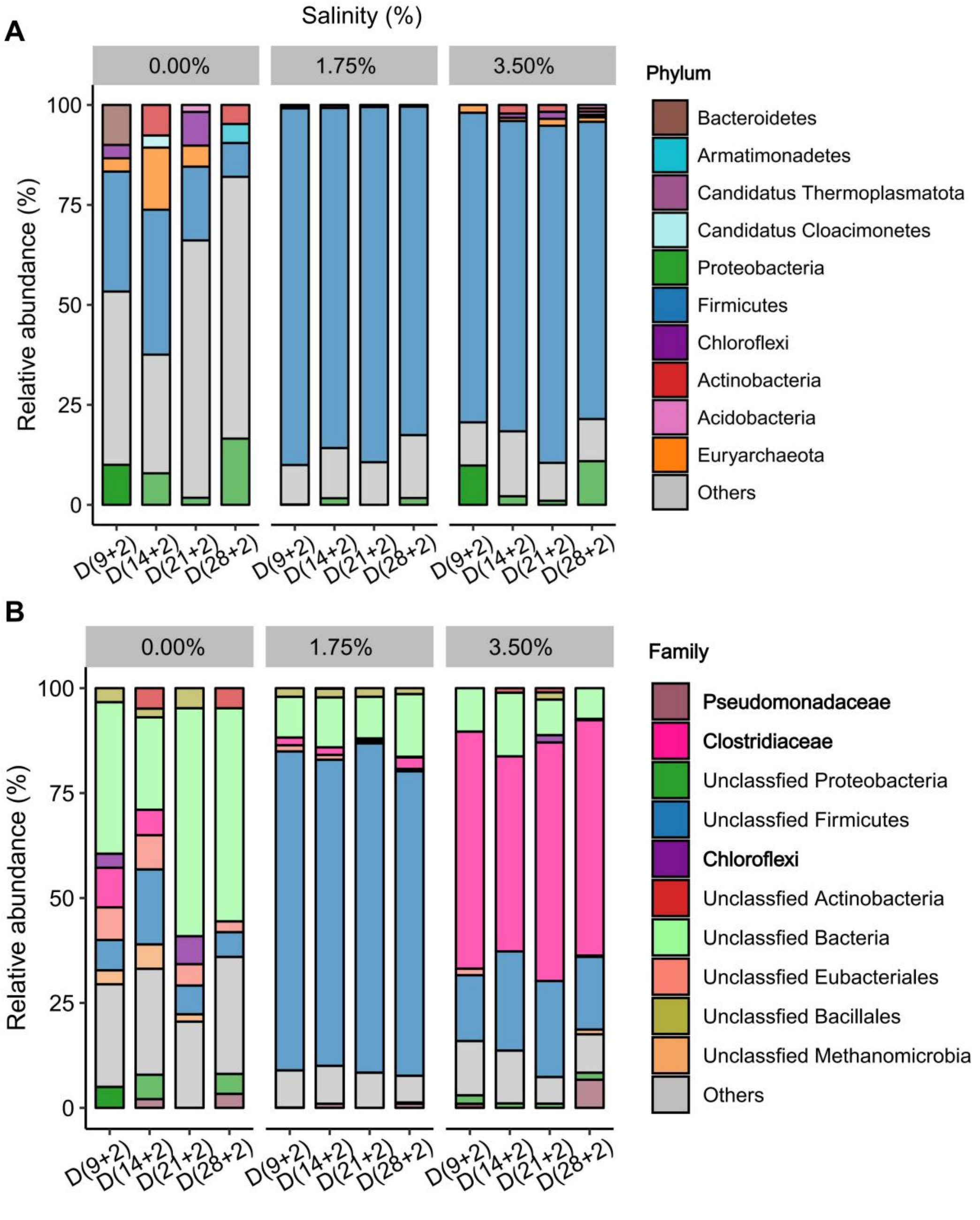

Figure S13 Taxonomic affiliation of transcripts associated with the module 'Stress Response: Osmotic Stress' at both the phylum level and the family level (top 10 taxa). The SEED IDs and the exact gene names of this module are listed in Table S9. The family level analysis also includes all higher-level unclassified taxa.

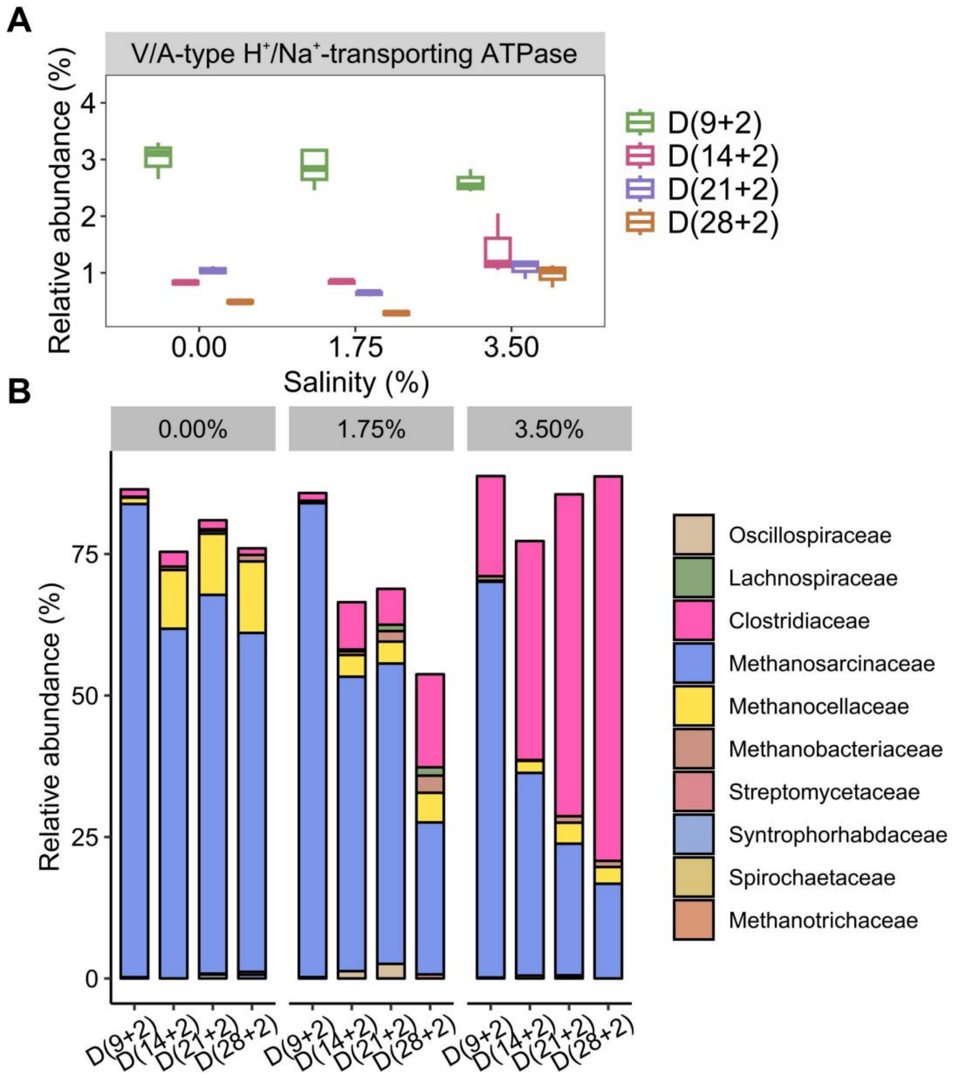

Figure S14 Differential abundance analysis of transcripts encoding V/A type H^+/Na^+-transporting ATPases and their taxonomic affiliations. The KEGG IDs and the exact gene names are listed in Table S9. (A) Relative abundance of transcripts encoding V/A type H^+/Na^+-transporting ATPases in response to preincubation time and salinity treatments, shown in relation to total metatranscriptomic mRNA. No significant differences were observed among the three salinity treatments; asterisks are not shown. (B) Taxonomic

composition of transcripts encoding V/A type H^+/Na^+-transporting ATPases at the family level (top 10) in response to preincubation time and salinity treatments.

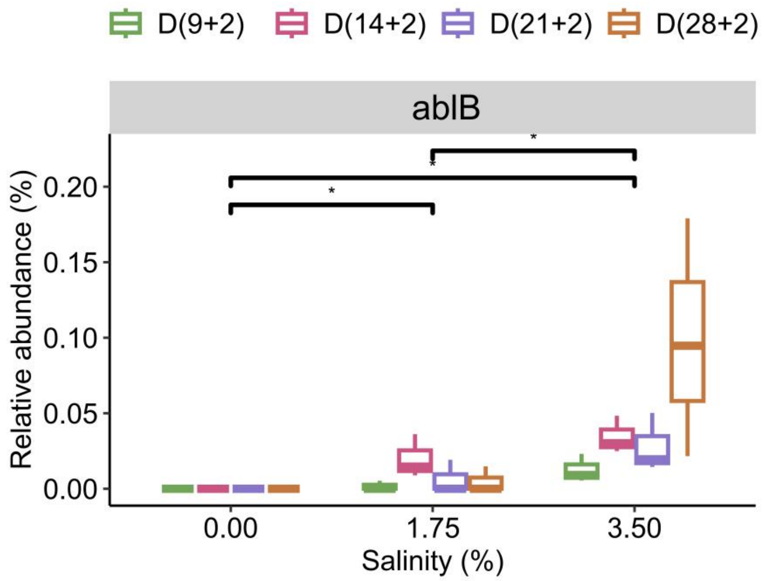

Figure S15 Relative abundance of *ablB* transcripts in response to preincubation time and salinity treatments, shown in relation to total metatranscriptomic mRNA. The *ablB* gene encodes the conversion of ß-lysine into the osmolyte N $^{\varepsilon}$–acetyl-ß-lysine. Asterisks indicate significant difference between the treatments (* P < 0.05, ** P < 0.01, *** P < 0.001).

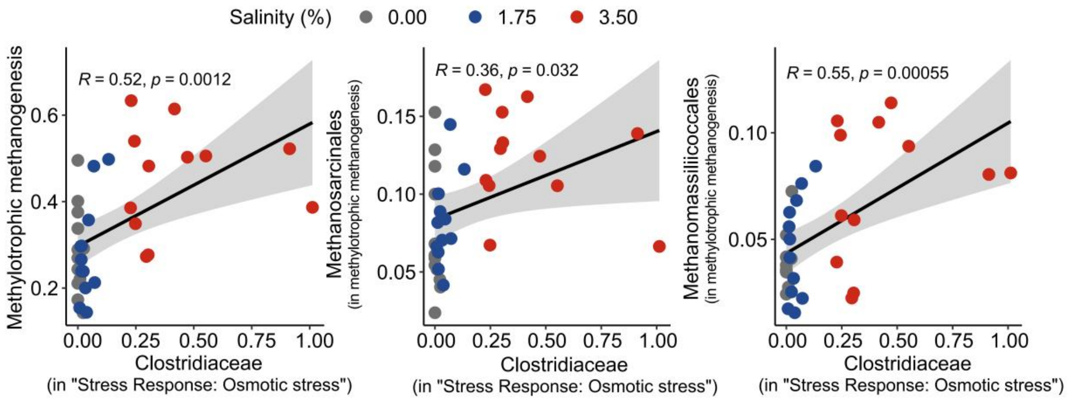

Figure S16 Abundance correlations of the *Clostridiaceae* transcripts assigned to osmotic stress (SEED level 3) with those encoding methylotrophic methanogenesis, methylotrophy by *Methanosarcinales*, and methylotrophy by *Methanomassiliicoccales*. The colors indicate the different salinity treatments. The "R" denotes the Pearson correlation coefficient, and the *P*-value is used to test the null hypothesis that there is no linear relationship between the two variables, n = 36. Note that *Methanomassiliicoccales* could not be annotated at the family level; therefore, correlations involving this group are presented at the order level.

Supplemental Tables

Table S1 Sequencing statistics of metatranscriptomic datasets. The sample identifiers C, M, and H represent the control (0.00%) treatment, as well as moderate (1.75%), and high (3.50%) salinity, respectively. The first two digits of the sample number indicate the total incubation period, including preincubation time and salinity exposure (days 9+2 [11]; 14+2 [16]; 21+2 [23]; 28+2 [30]). The last digit distinguishes between the triplicate samples of a given treatment.

Sample	Merged reads					mRNA						SSU rRNA			
	raw data	QC-minlen250	mean length	potential mRNA	%	nr BLAST hits	total	Bacteria	%	Archaea	%	Bacteria	%	Archaea	%
C111	5841253	1613826	300	87712	5.44	737799	33618	23758	70.67	9634	28.66	881953	54.65	143865	8.91
C112	3837685	1571219	310	58168	3.70	550510	24395	17357	71.15	6881	28.21	748626	47.65	117028	7.45
C113	4935749	1771485	304	73059	4.12	651675	28998	20025	69.06	8799	30.34	910063	51.37	148323	8.37
C161	4471932	1446148	296	48789	3.37	510327	23911	18672	78.09	5042	21.09	759276	52.50	155212	10.73
C162	4680086	1587610	300	49499	3.12	548801	25467	19879	78.06	5398	21.20	802360	50.54	157457	9.92
C163	4295407	1586940	302	50320	3.17	535700	24820	19528	78.68	5079	20.46	772584	48.68	148839	9.38
C231	4217125	1615884	306	54539	3.38	646877	28834	21716	75.31	6819	23.65	730592	45.21	132452	8.20
C232	3678228	1510170	307	45537	3.02	528658	23749	18351	77.27	5139	21.64	670415	44.39	108558	7.19
C233	5804136	1937314	300	68482	3.53	755404	34237	26049	76.08	7821	22.84	982010	50.69	173249	8.94

C301	4174218	1523765	303	55006	3.61	481090	23097	18302	79.24	4307	18.65	808688	53.07	156695	10.28
C302	3908117	1339909	299	51478	3.84	425895	20571	16412	79.78	3523	17.13	746381	55.70	143974	10.75
C303	5068017	1901497	303	65150	3.43	585411	27990	22213	79.36	5436	19.42	978519	51.46	217177	11.42
M111	9063165	3104470	303	79534	2.56	860297	38501	29614	76.92	8462	21.98	1576175	50.77	170078	5.48
M112	5639133	2175219	308	51450	2.37	656106	28672	19321	67.39	9163	31.96	1029302	47.32	165182	7.59
M113	6509785	2481031	307	52440	2.11	646569	28391	19884	70.04	8238	29.02	1205795	48.60	175928	7.09
M161	5744567	2285138	307	47072	2.06	492620	22393	18546	82.82	3505	15.65	1020290	44.65	155612	6.81
M162	6685657	2214739	301	38706	1.75	408193	19141	15197	79.40	3710	19.38	1127486	50.91	212723	9.60
M163	6308274	2201906	300	49843	2.26	502497	23642	18313	77.46	5127	21.69	1163960	52.86	218830	9.94
M231	4726215	1770153	306	37454	2.12	388613	17954	14732	82.05	2979	16.59	817704	46.19	139830	7.90
M232	6082416	1870765	298	43586	2.33	406453	19313	15677	81.17	3310	17.14	991209	52.98	151327	8.09
M233	6133707	2240099	304	47775	2.13	498553	23239	19108	82.22	3815	16.42	1059335	47.29	159504	7.12
M301	9507064	3395376	307	62226	1.83	540864	26249	21753	82.87	4047	15.42	1925055	56.70	311136	9.16
M302	11501415	4273223	305	66328	1.55	612229	28453	23747	83.46	4102	14.42	2303943	53.92	392381	9.18
M303	9033664	2714960	298	47202	1.74	365075	19048	15858	83.25	2883	15.14	1791003	65.97	317105	11.68
H111	9878318	3370471	303	58583	1.74	628224	28165	17493	62.11	10228	36.31	1568109	46.52	231695	6.87
H112	7253333	2792065	307	36313	1.30	373028	17794	12180	68.45	5335	29.98	1306816	46.80	143569	5.14
H113	8396603	2485703	300	41268	1.66	448562	20338	12146	59.72	7829	38.49	1367616	55.02	214139	8.61
H161	9688050	3701233	306	47734	1.29	383270	19291	13370	69.31	5497	28.50	1671984	45.17	318862	8.62
H162	8484220	3194370	304	41692	1.31	312232	16358	11994	73.32	4040	24.70	1483182	46.43	287035	8.99
H163	7547031	2431876	300	36507	1.50	253905	13526	10098	74.66	3158	23.35	1283897	52.79	208452	8.57

H231	9035960	3068802	306	45958	1.50	375401	19058	14470	75.93	4122	21.63	1443354	47.03	202633	6.60
H232	10527073	3947919	304	62606	1.59	513935	25615	19841	77.46	4882	19.06	1775105	44.96	221130	5.60
H233	7774416	2953621	308	36580	1.24	295823	15166	11752	77.49	3061	20.18	1313069	44.46	216625	7.33
H301	15343424	5680811	305	77107	1.36	575491	30308	23862	78.73	5825	19.22	2976293	52.39	664582	11.70
H302	14551485	4943846	302	41329	0.84	262289	13674	11183	81.78	2186	15.99	1235494	24.99	203398	4.11
H303	6499697	2419786	306	75264	3.11	513973	27109	22195	81.87	4381	16.16	2845729	117.60	427545	17.67

Table S2 Customized KEGG categories, including the list of genes (IDs) taken into consideration to analyze the relative expression level of each specific metabolic pathway.

Category	Pathway	ID
Central carbohydrate metabolism	Glycolysis	K00001, K00002, K00016, K00114, K00121, K00128, K00129, K00131, K00134, K00138, K00149, K00150, K00161, K00162, K00163, K00169, K00170, K00171, K00172, K00174, K00175, K00189, K00382, K00627, K00844, K00845, K00850, K00873, K00886, K00895, K00918, K00927, K01006, K01007, K01084, K01222, K01223, K01568, K01596, K01610, K01622, K01623, K01624, K01689, K01785, K01792, K01803, K01810, K01834, K01835, K01895, K01905, K02446, K02777, K02779, K02791, K03737, K03841, K04041, K04072, K06859, K10705, K11532, K11645, K12407, K12957, K13810, K13953, K13954, K13979, K14028, K14085, K15633, K15634, K15635, K15778, K15916, K16305, K16306, K16370, K18978, K20118, K20866, K21071, K22224, K24012, K25026
	Pyruvate metabolism	K00027, K00029, K00049, K00101, K00102, K00132, K00156, K00158, K00467, K00625, K00626, K00656, K00925, K01003, K01026, K01067, K01069, K01512, K01571, K01573, K01595, K01638, K01649, K01655, K01759, K01961, K01962, K01963, K02160, K02594, K03777, K03778, K04021, K05523, K10977, K11262, K11263, K12972, K13788, K15024, K18366, K18472, K18930, K19266, K20370, K20509, K21618, K22211, K22373
Fatty acid metabolism	Acetatogenesis	K00163, K00627, K00169, K03737, K00174, K00656, K13788, K04020, K01512, K00925
	Ethanol fermentation	K00163, K00627, K00169, K03737, K00174, K00656, K00132, K01568, K13951, K14028, K00114, K00002, K04022
	Propionate fermentation	K01595, K00024, K01676, K00239, K00244, K18209, K18556, K01899, K01902, K01847, K05606, K11264, K03416
Methane	Acetoclastic	K00925, K00625, K01895, K00193, K00197, K00194

metabolism	methanogenesis	
	Hydrogenotrophic methanogenesis	K00200, K00201, K00202, K00203, K11261, K00205, K11260, K00204, K00672, K01499, K13942, K00320, K00319
	Methylotrophic methanogenesis	K14080, K04480, K14081, K14082, K16177, K16176, K16179, K16178, K14084, K14083

Table S3 Influence of experimental factors and their interactions on the total microbial community, the bacterial community, and the methanogen community at the 16S rRNA level using PerMANOVA.

Total 16S rRNA			
salinity	$R2 = 0.251$	$F2,24 = 13.812$	$P = 0.001$
day	$R2 = 0.442$	$F3,24 = 16.174$	$P = 0.001$
salinity × day	$R2 = 0.088$	$F6,24 = 1.618$	$P = 0.028$
Bacterial 16S rRNA			
salinity	$R2 = 0.273$	$F2,24 = 15.449$	$P = 0.001$
day	$R2 = 0.418$	$F3,24 = 15.757$	$P = 0.001$
salt × day	$R2 = 0.096$	$F6,24 = 1.812$	$P = 0.007$
Methanogens 16S rRNA			
salinity	$R2 = 0.109$	$F2,24 = 5.079$	$P = 0.002$
day	$R2 = 0.598$	$F3,24 = 18.628$	$P = 0.001$
salinity × day	$R2 = 0.036$	$F6,24 = 0.558$	$P = 0.904$

Table S4 Influence of experimental factors and their interaction on the total microbial community, the bacterial community, and the methanogen community at the mRNA level using PerMANOVA.

Total mRNA			
salinity	$R^2 = 0.330$	$F_{2,24} = 15.262$	$P = 0.001$
day	$R^2 = 0.288$	$F_{3,24} = 8.881$	$P = 0.001$
salinity × day	$R^2 = 0.123$	$F_{6,24} = 1.896$	$P = 0.002$
**Bacterial mRNA**			
salinity	$R^2 = 0.427$	$F_{2,24} = 18.322$	$P = 0.001$
day	$R^2 = 0.140$	$F_{3,24} = 3.997$	$P = 0.001$
salinity × day	$R^2 = 0.153$	$F_{6,24} = 2.191$	$P = 0.003$
**Methanogen mRNA**			
salinity	$R^2 = 0.047$	$F_{2,24} = 2.837$	$P = 0.029$
day	$R^2 = 0.625$	$F_{3,24} = 25.13$	$P = 0.001$
salinity × day	$R^2 = 0.13$	$F_{6,24} = 2.608$	$P = 0.003$

Table S5 LEfSe biomarkers and their linear discriminant analysis (LDA) scores and Kruskal-Wallis (KW) values under the different salinity treatments on the SSU rRNA level. The sample identifiers C, M, and H represent the control (0.00%) treatment, as well as moderate (1.75%), and high (3.50%) salinity, respectively. The first two digits of the sample number indicate the total incubation period, including preincubation time and salinity exposure (days 9+2 [11]; 14+2 [16]; 21+2 [23]; 28+2 [30]). The last digit distinguishes between the triplicate samples of a given treatment.

Biomarker names	Enriched Groups	Log_{10} Average Abundance	LDA	KW_Pvalue
Bacteria; Firmicutes; Clostridia; Clostridiales; FamilyXVIII; uncultured; uncultured bacterium	C11	5.09	4.74	0.04
Bacteria; Firmicutes; Clostridia; Clostridiales; Christensenellaceae; Christensenellaceae R_7group; uncultured bacterium	M11	4.76	4.32	0.03
Bacteria; Firmicutes; Clostridia; Clostridiales; Clostridiaceae1; Clostridium sensustricto18	H11	4.99	4.69	0.03
Bacteria; Actinobacteria; Actinobacteria; Micrococcales; Intrasporangiaceae	H11	4.52	3.86	0.04
Bacteria; Tenericutes; Mollicutes; NB1_n; uncultured bacterium; uncultured bacterium; uncultured bacterium	M11	4.47	3.99	0.03
Bacteria; Firmicutes; Clostridia; Clostridiales; Ruminococcaceae; Ruminococcaceae UCG_010; uncultured bacterium	C11	4.43	4.09	0.04
Bacteria; Firmicutes; Clostridia; Clostridiales; Clostridiaceae1; Clostridium sensustricto18; Clostridium sporogenes	H11	4.72	4.45	0.03
Bacteria; Ignavibacteriae; Ignavibacteria; Ignavibacteriales; BSV40	C11	4.24	3.61	0.03
Bacteria; Firmicutes; Clostridia; Clostridiales; Lachnospiraceae; Mobilitalea; uncultured bacterium	M11	4.20	3.40	0.04
Bacteria; Firmicutes; Clostridia; Clostridiales; ClostridialesvadinBB60group; uncultured bacterium; uncultured bacterium	M11	4.25	3.81	0.03

Bacteria; Firmicutes; Clostridia; Clostridiales; Caldicoprobacteraceae; Caldicoprobacter; uncultured bacterium	M11	4.15	3.71	0.03
Bacteria; Firmicutes; Clostridia; Clostridiales; Peptococcaceae; uncultured; uncultured bacterium	C11	4.16	3.71	0.03
Bacteria; Firmicutes; Clostridia; Clostridiales; Ruminococcaceae; uncultured	C11	4.06	3.55	0.04
Bacteria; Firmicutes; Clostridia; Clostridiales; FamilyXIII; uncultured; uncultured bacterium	M11	4.10	3.79	0.04
Bacteria; Firmicutes; Clostridia; Clostridiales; Christensenellaceae; uncultured; uncultured bacterium	M11	4.06	3.61	0.04
Bacteria; Firmicutes; Clostridia; Clostridiales; Ruminococcaceae; Ruminiclostridium; uncultured bacterium	M11	4.04	3.58	0.03
Archaea; Euryarchaeota; Methanobacteria; Methanobacteriales; Methanobacteriaceae; Methanobacterium_Ambiguous_taxa	H11	4.00	3.56	0.04
Bacteria; Actinobacteria; Actinobacteria; Micrococcales; Intrasporangiaceae; Phycicoccus	H11	3.95	3.55	0.04
Bacteria; Firmicutes; Clostridia; Clostridiales; Christensenellaceae; ChristensenellaceaeR_7group	M11	4.01	3.87	0.03
Bacteria; Actinobacteria; Actinobacteria; Propionibacteriales; Nocardioidaceae; Marmoricola	H11	3.94	3.75	0.03
Bacteria; Ignavibacteriae; Ignavibacteria; Ignavibacteriales; Ignavibacteriaceae; Ignavibacterium; uncultured bacterium	C11	3.91	3.32	0.03
Bacteria; Firmicutes; Clostridia; Clostridiales; Caldicoprobacteraceae; Caldicoprobacter	M11	3.95	3.65	0.04
Bacteria; Firmicutes; Clostridia; Clostridiales; FamilyXVIII	C11	5.09	4.71	0.04
Bacteria; Firmicutes; Clostridia; Clostridiales; FamilyXVIII; uncultured	C11	5.09	4.71	0.04
Bacteria; Firmicutes; Clostridia; Clostridiales; Christensenellaceae	M11	4.94	4.40	0.03
Bacteria; Actinobacteria; Actinobacteria; Micrococcales	H11	4.62	4.03	0.04
Bacteria; Tenericutes	M11	4.47	4.00	0.03
Bacteria; Tenericutes; Mollicutes	M11	4.47	4.01	0.03
Bacteria; Tenericutes; Mollicutes; NB1_n	M11	4.47	3.96	0.03
Bacteria; Tenericutes; Mollicutes; NB1_n; uncultured bacterium	M11	4.47	4.03	0.03
Bacteria; Tenericutes; Mollicutes; NB1_n; uncultured bacterium; uncultured bacterium	M11	4.47	3.98	0.03

Bacteria; Firmicutes; Clostridia; Clostridiales; Ruminococcaceae; Ruminococcaceae UCG_010	C11	4.43	4.07	0.04
Bacteria; Ignavibacteriae	C11	4.41	3.71	0.03
Bacteria; Ignavibacteriae; Ignavibacteria	C11	4.41	3.71	0.03
Bacteria; Ignavibacteriae; Ignavibacteria; Ignavibacteriales	C11	4.41	3.78	0.03
Bacteria; Firmicutes; Clostridia; Clostridiales; Clostridiales vadinBB60 group	M11	4.25	3.88	0.03
Bacteria; Firmicutes; Clostridia; Clostridiales; Clostridiales vadinBB60 group; uncultured bacterium	M11	4.25	3.84	0.03
Bacteria; Firmicutes; Clostridia; Clostridiales; Caldicoprobacteraceae	M11	4.36	3.85	0.03
Bacteria; Firmicutes; Clostridia; Clostridiales; Peptococcaceae; uncultured	C11	4.16	3.70	0.03
Bacteria; Firmicutes; Clostridia; Clostridiales; FamilyXIII	M11	4.10	3.66	0.04
Bacteria; Firmicutes; Clostridia; Clostridiales; FamilyXIII; uncultured	M11	4.10	3.68	0.04
Bacteria; Firmicutes; Clostridia; Clostridiales; Ruminococcaceae; Ruminiclostridium	M11	4.04	3.74	0.03
Archaea; Euryarchaeota; Methanobacteria	H11	4.00	3.53	0.04
Archaea; Euryarchaeota; Methanobacteria; Methanobacteriales	H11	4.00	3.53	0.04
Archaea; Euryarchaeota; Methanobacteria; Methanobacteriales; Methanobacteriaceae	H11	4.00	3.53	0.04
Bacteria; Ignavibacteriae; Ignavibacteria; Ignavibacteriales; Ignavibacteriaceae	C11	3.91	3.46	0.03
Bacteria; Ignavibacteriae; Ignavibacteria; Ignavibacteriales; Ignavibacteriaceae; Ignavibacterium	C11	3.91	3.40	0.03
Bacteria; Firmicutes; Clostridia; Clostridiales; FamilyXVIII; uncultured; uncultured bacterium	C16	4.84	4.48	0.03
Bacteria; Proteobacteria; Deltaproteobacteria; Myxococcales; Haliangiaceae; Haliangium; uncultured bacterium	H16	4.57	3.77	0.04
Bacteria; Cyanobacteria; Cyanobacteria; SubsectionIV; FamilyI_Ambiguous_taxa_Ambiguous_taxa	H16	4.53	3.76	0.04
Bacteria; Firmicutes; Clostridia; Clostridiales; Ruminococcaceae; Ruminiclostridium1; uncultured bacterium	C16	4.59	3.97	0.03
Bacteria; Firmicutes; Clostridia; Clostridiales; Clostridiaceae1; Clostridium sensustricto18	H16	4.87	4.54	0.03

Bacteria; Firmicutes; Clostridia; Clostridiales; Ruminococcaceae; uncultured; uncultured bacterium	C16	4.45	3.66	0.04
Bacteria; Actinobacteria; Thermoleophilia; Gaiellales; uncultured; uncultured bacterium; uncultured bacterium	H16	4.39	3.66	0.04
Bacteria; Firmicutes; Clostridia; Clostridiales; Clostridiaceae1; Fonticella	C16	4.45	3.93	0.03
Bacteria; Proteobacteria; Deltaproteobacteria; Myxococcales; Polyangiaceae; Sorangium; uncultured bacterium	H16	4.32	3.53	0.04
Bacteria; Firmicutes; Clostridia; Clostridiales; Ruminococcaceae	C16	4.33	3.59	0.03
Bacteria; Firmicutes; Clostridia; Clostridiales; Clostridiaceae1; Fonticella_Ambiguous_taxa	C16	4.36	3.84	0.03
Bacteria; Firmicutes; Clostridia; Clostridiales; Clostridiaceae1; Clostridium sensustricto18; Clostridium sporogenes	H16	4.63	4.29	0.03
Bacteria; Firmicutes; Clostridia; Clostridiales; Ruminococcaceae; RuminococcaceaeUCG_010; uncultured bacterium	C16	4.35	3.94	0.03
Bacteria; Firmicutes; Clostridia; Clostridiales; Clostridiales vadinBB60 group; uncultured bacterium; uncultured bacterium	M16	4.20	3.60	0.04
Bacteria; Chloroflexi; SJA_15; uncultured bacterium; uncultured bacterium; uncultured bacterium; uncultured bacterium	H16	4.18	3.49	0.04
Archaea; Euryarchaeota; Methanomicrobia; Methanosarcinales; Methanosaetaceae; Methanosaeta; uncultured archaeon	H16	4.13	3.39	0.04
Bacteria; Planctomycetes; Planctomycetacia; Planctomycetales; Planctomycetaceae	H16	4.06	3.21	0.04
Bacteria; Firmicutes; Clostridia; Clostridiales; Gracilibacteraceae; Lutispora; uncultured bacterium	C16	4.08	3.57	0.03
Bacteria; Proteobacteria; Deltaproteobacteria; Myxococcales; Polyangiaceae	H16	4.09	3.48	0.04
Bacteria; Firmicutes; Clostridia; Clostridiales; Christensenellaceae; ChristensenellaceaeR_7group	M16	4.11	3.67	0.03
Bacteria; Firmicutes; Clostridia; Clostridiales; Ruminococcaceae; uncultured	C16	3.99	3.43	0.04
Bacteria; Firmicutes; Limnochordia; Limnochordales; Limnochordaceae	M16	3.85	3.06	0.03
Bacteria; Actinobacteria; Thermoleophilia; Gaiellales; uncultured	H16	3.84	3.33	0.04
Bacteria; Actinobacteria; Acidimicrobiia; Acidimicrobiales; uncultured	H16	3.82	3.52	0.04
Archaea; Thaumarchaeota; SoilCrenarchaeoticGroup_SCG_	H16	3.78	3.41	0.03
Bacteria; Actinobacteria; Actinobacteria; Corynebacteriales; Mycobacteriaceae; Mycobacterium_Ambiguous_taxa	H16	3.77	3.04	0.04

Bacteria; Firmicutes; Clostridia; Clostridiales; Ruminococcaceae; Ruminococcaceae UCG_010	C16	3.98	3.68	0.03
Bacteria; Firmicutes; Clostridia; Clostridiales; Christensenellaceae	M16	5.00	4.45	0.03
Bacteria; Firmicutes; Clostridia; Clostridiales; FamilyXVIII	C16	4.84	4.46	0.03
Bacteria; Firmicutes; Clostridia; Clostridiales; FamilyXVIII; uncultured	C16	4.84	4.46	0.03
Bacteria; Cyanobacteria	H16	4.71	3.83	0.04
Bacteria; Actinobacteria; Thermoleophilia; Gaiellales	H16	4.59	3.87	0.04
Bacteria; Actinobacteria; Thermoleophilia; Gaiellales; uncultured; uncultured bacterium	H16	4.39	3.73	0.04
Bacteria; Firmicutes; Clostridia; Clostridiales; Clostridiales vadinBB60 group	M16	4.20	3.49	0.04
Bacteria; Firmicutes; Clostridia; Clostridiales; Clostridiales vadinBB60 group; uncultured bacterium	M16	4.20	3.59	0.04
Bacteria; Chloroflexi; SJA_15; uncultured bacterium	H16	4.18	3.51	0.04
Bacteria; Chloroflexi; SJA_15; uncultured bacterium; uncultured bacterium	H16	4.18	3.61	0.04
Bacteria; Chloroflexi; SJA_15; uncultured bacterium; uncultured bacterium; uncultured bacterium	H16	4.18	3.56	0.04
Archaea; Euryarchaeota; Methanomicrobia; Methanosarcinales; Methanosaetaceae	H16	4.13	3.37	0.04
Archaea; Euryarchaeota; Methanomicrobia; Methanosarcinales; Methanosaetaceae; Methanosaeta	H16	4.13	3.39	0.04
Bacteria; Firmicutes; Clostridia; Clostridiales; Gracilibacteraceae	C16	4.08	3.52	0.03
Bacteria; Firmicutes; Clostridia; Clostridiales; Gracilibacteraceae; Lutispora	C16	4.08	3.50	0.03
Bacteria; Actinobacteria; Acidimicrobiia	H16	4.11	3.43	0.04
Bacteria; Actinobacteria; Acidimicrobiia; Acidimicrobiales	H16	4.11	3.43	0.04
Bacteria; Actinobacteria; Actinobacteria; Corynebacteriales	H16	3.77	3.29	0.04
Bacteria; Actinobacteria; Actinobacteria; Corynebacteriales; Mycobacteriaceae	H16	3.77	3.32	0.04
Bacteria; Firmicutes; Clostridia; Clostridiales; Clostridiaceae1; Clostridium sensustricto10	H23	5.21	4.51	0.04

Bacteria; Firmicutes; Clostridia; Clostridiales; Clostridiaceae1; Clostridium sensustricto10; uncultured bacterium	H23	4.98	4.28	0.03
Bacteria; Firmicutes; Clostridia; Clostridiales; Clostridiaceae1; Clostridium sensustricto10_Ambiguous_taxa	H23	4.90	4.15	0.03
Bacteria; Firmicutes; Clostridia; Clostridiales; Clostridiaceae1; Clostridium sensustricto8; uncultured bacterium	H23	4.90	4.15	0.03
Bacteria; Firmicutes; Clostridia; Clostridiales; Clostridiaceae1; Clostridium sensustricto8	H23	4.89	4.22	0.04
Archaea; Euryarchaeota; Methanomicrobia; Methanocellales; Methanocellaceae; Methanocella; uncultured archaeon	H23	4.74	3.73	0.03
Bacteria; Firmicutes; Clostridia; Clostridiales; Clostridiaceae1; Clostridium sensustricto18	H23	5.09	4.77	0.03
Bacteria; Firmicutes; Clostridia; Clostridiales; FamilyXVIII; uncultured; uncultured bacterium	C23	4.76	4.44	0.03
Bacteria; Firmicutes; Clostridia; Clostridiales; Clostridiaceae1; Clostridium sensustricto8_Ambiguous_taxa	H23	4.63	3.96	0.03
Bacteria; Actinobacteria; Actinobacteria; Propionibacteriales; Nocardioidaceae; Nocardioides	H23	4.56	3.67	0.03
Bacteria; Ignavibacteriae; Ignavibacteria; Ignavibacteriales; BSV40	C23	4.58	4.01	0.04
Bacteria; Firmicutes; Clostridia; Clostridiales; Ruminococcaceae; Ruminiclostridium1; uncultured bacterium	C23	4.58	3.91	0.03
Bacteria; Firmicutes; Clostridia; Clostridiales; Clostridiaceae1; Clostridium sensustricto18; Clostridium sporogenes	H23	4.82	4.53	0.03
Bacteria; Acidobacteria; Solibacteres; Solibacterales; Solibacteraceae_Subgroup3_; Candidatus Solibacter	C23	4.39	3.76	0.03
Bacteria; Proteobacteria; Deltaproteobacteria; Myxococcales; Polyangiaceae; Sorangium; uncultured bacterium	H23	4.34	3.70	0.04
Bacteria; Actinobacteria; Thermoleophilia; Gaiellales; uncultured; uncultured bacterium; uncultured bacterium	H23	4.32	3.64	0.04
Bacteria; Firmicutes; Clostridia; Clostridiales; Heliobacteriaceae; Hydrogenispora; uncultured bacterium	C23	4.34	3.74	0.04
Bacteria; Firmicutes; Clostridia; Clostridiales; Lachnospiraceae; Anaerosporobacter	H23	4.34	3.83	0.03
Bacteria; Firmicutes; Clostridia; Clostridiales; Peptococcaceae; uncultured; uncultured bacterium	C23	4.28	3.69	0.03
Bacteria; Firmicutes; Clostridia; Clostridiales; Ruminococcaceae; RuminococcaceaeUCG_010; uncultured bacterium	C23	4.24	3.71	0.03
Bacteria; Acidobacteria; Acidobacteria; Acidobacteriales; Acidobacteriaceae_Subgroup1_; uncultured	C23	4.22	3.68	0.03
Bacteria; Firmicutes; Clostridia; Clostridiales; Christensenellaceae; uncultured; uncultured bacterium	M23	4.18	3.61	0.04

Bacteria; Acidobacteria; Solibacteres; Solibacterales; Solibacteraceae_Subgroup3_; Candidatus Solibacter; uncultured bacterium	C23	4.16	3.55	0.04
Bacteria; Firmicutes; Clostridia; Clostridiales; Clostridiaceae1; Fonticella_Ambiguous_taxa	C23	4.13	3.47	0.03
Bacteria; Firmicutes; Clostridia; Clostridiales; Clostridiaceae1; Fonticella	C23	4.15	3.64	0.03
Bacteria; Proteobacteria; Deltaproteobacteria; Myxococcales; Polyangiaceae	H23	4.13	3.62	0.03
Bacteria; Cyanobacteria; ML635J_21; uncultured bacterium; uncultured bacterium; uncultured bacterium; uncultured bacterium	H23	4.12	3.53	0.04
Bacteria; Actinobacteria; Actinobacteria; Propionibacteriales; Nocardioidaceae; Nocardioides; uncultured bacterium	H23	4.07	3.37	0.03
Bacteria; Proteobacteria; Alphaproteobacteria; Rhizobiales; Bradyrhizobiaceae	H23	4.11	3.53	0.03
Bacteria; Firmicutes; Clostridia; Clostridiales; Christensenellaceae; uncultured	M23	4.05	3.42	0.03
Bacteria; Firmicutes; Clostridia; Clostridiales; Clostridiaceae1; Clostridium sensustricto9; uncultured bacterium	H23	4.04	3.49	0.03
Archaea; Euryarchaeota; Methanomicrobia; Methanocellales; Methanocellaceae; Rice ClusterI; uncultured archaeon	H23	3.98	3.33	0.03
Bacteria; Proteobacteria; Deltaproteobacteria; Myxococcales; Sandaracinaceae; uncultured; uncultured bacterium	H23	3.99	3.27	0.03
Bacteria; Firmicutes; Bacilli; Bacillales; Paenibacillaceae; Paenibacillus	H23	3.98	3.44	0.03
Bacteria; Firmicutes; Clostridia; Clostridiales; Lachnospiraceae; Anaerostipes; uncultured bacterium	H23	4.05	3.64	0.03
Bacteria; Actinobacteria; Thermoleophilia; Solirubrobacterales	H23	3.93	3.37	0.03
Bacteria; Proteobacteria; Deltaproteobacteria; Desulfuromonadales; Geobacteraceae; Geobacter; uncultured bacterium	M23	4.06	3.61	0.03
Bacteria; Firmicutes; Clostridia; Clostridiales; Peptococcaceae; Desulfotomaculum	C23	3.90	3.24	0.03
Bacteria; Chloroflexi; Anaerolineae; Anaerolineales; Anaerolineaceae; uncultured; uncultured bacterium	M23	3.92	3.40	0.03
Bacteria; Firmicutes; Clostridia; Clostridiales; Clostridiaceae1; Clostridium sensustricto 9	H23	3.90	3.36	0.04
Bacteria; Proteobacteria; Deltaproteobacteria; Myxococcales; Polyangiaceae; Sorangium	H23	3.86	3.27	0.03
Bacteria; Elusimicrobia; Elusimicrobia; LineageI; Unknown Family; Candidatus Endomicrobium; uncultured bacterium	C23	3.94	3.53	0.03
Bacteria; Firmicutes; Clostridia; Clostridiales; Peptococcaceae; Sporotomaculum; uncultured bacterium	C23	3.88	3.46	0.03

Bacteria; Proteobacteria; Deltaproteobacteria; Bdellovibrionales; Bdellovibrionaceae; OM27clade; uncultured bacterium	H23	3.82	3.36	0.04
Archaea; Euryarchaeota; Methanomicrobia; Methanocellales; Methanocellaceae; Methanocella	H23	4.74	3.76	0.03
Bacteria; Firmicutes; Clostridia; Clostridiales; FamilyXVIII	C23	4.76	4.44	0.03
Bacteria; Firmicutes; Clostridia; Clostridiales; FamilyXVIII; uncultured	C23	4.76	4.43	0.03
Bacteria; Actinobacteria; Actinobacteria; Propionibacteriales	H23	4.80	3.88	0.04
Bacteria; Actinobacteria; Actinobacteria; Propionibacteriales; Nocardioidaceae	H23	4.80	3.97	0.04
Bacteria; Ignavibacteriae	C23	4.73	4.14	0.04
Bacteria; Ignavibacteriae; Ignavibacteria	C23	4.73	4.05	0.04
Bacteria; Ignavibacteriae; Ignavibacteria; Ignavibacteriales	C23	4.73	4.08	0.04
Bacteria; Acidobacteria; Solibacteres	C23	4.59	3.91	0.03
Bacteria; Acidobacteria; Solibacteres; Solibacterales	C23	4.59	3.91	0.03
Bacteria; Acidobacteria; Solibacteres; Solibacterales; Solibacteraceae_Subgroup3_	C23	4.59	3.88	0.03
Bacteria; Actinobacteria; Thermoleophilia	H23	4.47	3.77	0.04
Bacteria; Actinobacteria; Thermoleophilia; Gaiellales	H23	4.32	3.64	0.04
Bacteria; Actinobacteria; Thermoleophilia; Gaiellales; uncultured	H23	4.32	3.71	0.04
Bacteria; Actinobacteria; Thermoleophilia; Gaiellales; uncultured; uncultured bacterium	H23	4.32	3.77	0.04
Bacteria; Firmicutes; Clostridia; Clostridiales; Heliobacteriaceae	C23	4.34	3.80	0.04
Bacteria; Firmicutes; Clostridia; Clostridiales; Heliobacteriaceae; Hydrogenispora	C23	4.34	3.84	0.04
Bacteria; Firmicutes; Clostridia; Clostridiales; Peptococcaceae; uncultured	C23	4.28	3.69	0.03
Bacteria; Firmicutes; Clostridia; Clostridiales; Ruminococcaceae; Ruminococcaceae UCG_010	C23	4.24	3.70	0.03
Bacteria; Acidobacteria; Acidobacteria	C23	4.22	3.65	0.03

Bacteria; Acidobacteria; Acidobacteria; Acidobacteriales	C23	4.22	3.64	0.03
Bacteria; Acidobacteria; Acidobacteria; Acidobacteriales; Acidobacteriaceae_Subgroup1_	C23	4.22	3.71	0.03
Bacteria; Cyanobacteria; ML635J_21	H23	4.12	3.55	0.04
Bacteria; Cyanobacteria; ML635J_21; uncultured bacterium	H23	4.12	3.58	0.04
Bacteria; Cyanobacteria; ML635J_21; uncultured bacterium; uncultured bacterium	H23	4.12	3.50	0.04
Bacteria; Cyanobacteria; ML635J_21; uncultured bacterium; uncultured bacterium; uncultured bacterium	H23	4.12	3.54	0.04
Bacteria; Proteobacteria; Deltaproteobacteria; Myxococcales; Sandaracinaceae	H23	3.99	3.39	0.03
Bacteria; Proteobacteria; Deltaproteobacteria; Myxococcales; Sandaracinaceae; uncultured	H23	3.99	3.48	0.03
Bacteria; Firmicutes; Clostridia; Clostridiales; Lachnospiraceae; Anaerostipes	H23	4.05	3.64	0.03
Bacteria; Proteobacteria; Deltaproteobacteria; Desulfuromonadales	M23	4.06	3.69	0.03
Bacteria; Proteobacteria; Deltaproteobacteria; Desulfuromonadales; Geobacteraceae	M23	4.06	3.63	0.03
Bacteria; Proteobacteria; Deltaproteobacteria; Desulfuromonadales; Geobacteraceae; Geobacter	M23	4.06	3.65	0.03
Bacteria; Chloroflexi; Anaerolineae	M23	3.92	3.25	0.03
Bacteria; Chloroflexi; Anaerolineae; Anaerolineales	M23	3.92	3.31	0.03
Bacteria; Chloroflexi; Anaerolineae; Anaerolineales; Anaerolineaceae	M23	3.92	3.57	0.03
Bacteria; Chloroflexi; Anaerolineae; Anaerolineales; Anaerolineaceae; uncultured	M23	3.92	3.30	0.03
Bacteria; Elusimicrobia	C23	3.94	3.50	0.03
Bacteria; Elusimicrobia; Elusimicrobia	C23	3.94	3.47	0.03
Bacteria; Elusimicrobia; Elusimicrobia; LineageI	C23	3.94	3.54	0.03
Bacteria; Elusimicrobia; Elusimicrobia; LineageI; UnknownFamily	C23	3.94	3.53	0.03
Bacteria; Elusimicrobia; Elusimicrobia; LineageI; UnknownFamily; Candidatus Endomicrobium	C23	3.94	3.50	0.03

Bacteria; Firmicutes; Clostridia; Clostridiales; Peptococcaceae; Sporotomaculum	C23	3.88	3.40	0.03
Bacteria; Proteobacteria; Deltaproteobacteria; Bdellovibrionales	H23	3.82	3.60	0.04
Bacteria; Proteobacteria; Deltaproteobacteria; Bdellovibrionales; Bdellovibrionaceae	H23	3.82	3.40	0.04
Bacteria; Proteobacteria; Deltaproteobacteria; Bdellovibrionales; Bdellovibrionaceae; OM27clade	H23	3.82	3.29	0.04
Bacteria; Firmicutes; Clostridia; Clostridiales; Clostridiaceae1; Clostridium sensustricto18	H30	4.91	4.63	0.03
Bacteria; Firmicutes; Clostridia; Clostridiales; Clostridiaceae1; Clostridium sensustricto18; Clostridiumsporogenes	H30	4.63	4.38	0.03
Bacteria; Elusimicrobia; Elusimicrobia; LineageI; UnknownFamily; Candidatus Endomicrobium; uncultured bacterium	C30	4.13	3.48	0.04
Bacteria; Proteobacteria; Alphaproteobacteria; Rhizobiales; Bradyrhizobiaceae	H30	4.01	3.76	0.04
Bacteria; Elusimicrobia	C30	4.13	3.60	0.04
Bacteria; Elusimicrobia; Elusimicrobia	C30	4.13	3.68	0.04
Bacteria; Elusimicrobia; Elusimicrobia; LineageI	C30	4.13	3.58	0.04
Bacteria; Elusimicrobia; Elusimicrobia; LineageI; Unknown Family	C30	4.13	3.66	0.04
Bacteria; Elusimicrobia; Elusimicrobia; LineageI; Unknown Family; Candidatus Endomicrobium	C30	4.13	3.67	0.04

Table S6 LEfSe biomarkers and their linear discriminant analysis (LDA) scores and Kruskal-Wallis (KW) values under the different salinity treatments at the mRNA level. The sample identifiers C, M, and H represent the control (0.00%) treatment, as well as moderate (1.75%), and high (3.50%) salinity, respectively. The first two digits of the sample number indicate the total incubation period, including preincubation time and salinity exposure (days 9+2 [11]; 14+2 [16]; 21+2 [23]; 28+2 [30]). The last digit distinguishes between the triplicate samples of a given treatment.

Biomarker names	Enriched Groups	Log10 Average Abundance	LDA	KW_Pvalue
Bacteria; Firmicutes; Clostridia; Eubacteriales; Clostridiaceae; Clostridium; _Clostridium_	H11	5.29	4.93	0.04
Archaea; Euryarchaeota; Methanomicrobia; Methanosarcinales; Methanosarcinaceae; Methanosarcina; Methanosarcinaspelaei	C11	4.69	4.18	0.04
Archaea; Euryarchaeota; Methanomicrobia; Methanosarcinales; Methanosarcinaceae; Methanosarcina; Methanosarcinasp_Ant1	M11	4.56	3.92	0.03
Bacteria; Firmicutes; Clostridia; Eubacteriales; Clostridiaceae; Oxobacter; Oxobacterpfennigii	C11	4.68	4.22	0.03
Bacteria; Firmicutes; Clostridia; Eubacteriales; Symbiobacteriaceae; Symbiobacterium; _Symbiobacterium_	C11	4.33	4.05	0.03
Bacteria; Firmicutes; Clostridia; Eubacteriales; Clostridiaceae; Fonticella; Fonticellatunisiensis	C11	4.22	3.91	0.03
Bacteria; Firmicutes; Clostridia; Eubacteriales; Symbiobacteriaceae; Symbiobacterium; Symbiobacteriumterraclitae	C11	4.23	3.90	0.03
Bacteria; Firmicutes; Bacilli; Bacillales; Paenibacillaceae; Paenibacillus; _Paenibacillus_	C11	4.12	3.70	0.04
Bacteria; Firmicutes; Clostridia; Eubacteriales; Peptococcaceae; Desulfitobacterium; Desulfitobacteriummetallireducens	C11	4.00	3.69	0.03
Bacteria; Firmicutes; Clostridia; Eubacteriales; Peptococcaceae; _Peptococcaceae_; _Peptococcaceae_	C11	3.91	3.56	0.04

Bacteria; Proteobacteria; Gammaproteobacteria; Pseudomonadales; Pseudomonadaceae; Pseudomonas; _Pseudomonas_	H11	3.82	3.48	0.03
Archaea; Euryarchaeota; Methanomicrobia; Methanocellales; Methanocellaceae; Methanocella; Methanocellasp_PtaU1_Bin125	C11	3.85	3.53	0.04
Bacteria; Firmicutes; Bacilli; Bacillales; Paenibacillaceae; _Paenibacillaceae_; _Paenibacillaceae_	C11	3.78	3.64	0.03
Eukaryota; Discosea; _Longamoebia_; Longamoebia; Acanthamoebidae; Acanthamoeba; Acanthamoebacastellanii	H11	3.68	3.42	0.04
Bacteria; Actinobacteria; Actinomycetia; Propionibacteriales; Nocardioidaceae; _Nocardioidaceae_; _Nocardioidaceae_	H11	3.67	3.54	0.03
Bacteria; Firmicutes; Negativicutes; Selenomonadales; Sporomusaceae; Anaerosporomusa; Anaerosporomusasubterranea	C11	3.63	3.57	0.03
Bacteria; Firmicutes; Clostridia; Eubacteriales; Oscillospiraceae; Sporobacter; Sporobactertermitidis	C11	3.53	3.37	0.04
Bacteria; Firmicutes; Clostridia; Eubacteriales; Clostridiaceae	H11	5.48	4.87	0.03
Bacteria; Firmicutes; Clostridia; Eubacteriales; Clostridiaceae; Oxobacter	C11	4.68	4.25	0.03
Bacteria; Firmicutes; Clostridia; Eubacteriales; Symbiobacteriaceae	C11	4.67	4.36	0.03
Bacteria; Firmicutes; Clostridia; Eubacteriales; Symbiobacteriaceae; Symbiobacterium	C11	4.67	4.36	0.03
Bacteria; Firmicutes; Clostridia; Eubacteriales; Clostridiaceae; Fonticella	C11	4.22	3.93	0.03
Bacteria; Firmicutes; Bacilli; Bacillales; Paenibacillaceae	C11	4.28	3.90	0.03
Bacteria; Firmicutes; Bacilli; Bacillales; Paenibacillaceae; Paenibacillus	C11	4.12	3.69	0.04
Bacteria; Firmicutes; Clostridia; Eubacteriales; Peptococcaceae	C11	4.41	3.95	0.03
Bacteria; Firmicutes; Clostridia; Eubacteriales; Peptococcaceae; Desulfitobacterium	C11	4.00	3.71	0.03
Bacteria; Firmicutes; Clostridia; Eubacteriales; Peptococcaceae; _Peptococcaceae_	C11	3.91	3.58	0.04
Bacteria; Proteobacteria; Gammaproteobacteria	H11	4.26	3.81	0.04
Bacteria; Proteobacteria; Gammaproteobacteria; Pseudomonadales	H11	3.82	3.48	0.03
Bacteria; Proteobacteria; Gammaproteobacteria; Pseudomonadales; Pseudomonadaceae	H11	3.82	3.51	0.03
Bacteria; Proteobacteria; Gammaproteobacteria; Pseudomonadales; Pseudomonadaceae; Pseudomonas	H11	3.82	3.59	0.03

Bacteria; Firmicutes; Bacilli; Bacillales; Paenibacillaceae; _Paenibacillaceae_	C11	3.78	3.58	0.03
Eukaryota	H11	3.68	3.29	0.04
Eukaryota; Discosea	H11	3.68	3.33	0.04
Eukaryota; Discosea; _Longamoebia_	H11	3.68	3.34	0.04
Eukaryota; Discosea; _Longamoebia_; Longamoebia	H11	3.68	3.34	0.04
Eukaryota; Discosea; _Longamoebia_; Longamoebia; Acanthamoebidae	H11	3.68	3.32	0.04
Eukaryota; Discosea; _Longamoebia_; Longamoebia; Acanthamoebidae; Acanthamoeba	H11	3.68	3.38	0.04
Bacteria; Firmicutes; Negativicutes	C11	4.04	3.70	0.03
Bacteria; Firmicutes; Negativicutes; Selenomonadales	C11	4.04	3.72	0.03
Bacteria; Firmicutes; Negativicutes; Selenomonadales; Sporomusaceae	C11	4.04	3.71	0.03
Bacteria; Actinobacteria	H11	3.90	3.63	0.03
Bacteria; Actinobacteria; Actinomycetia	H11	3.90	3.69	0.03
Bacteria; Actinobacteria; Actinomycetia; Propionibacteriales	H11	3.90	3.65	0.03
Bacteria; Actinobacteria; Actinomycetia; Propionibacteriales; Nocardioidaceae	H11	3.90	3.62	0.03
Bacteria; Actinobacteria; Actinomycetia; Propionibacteriales; Nocardioidaceae; _Nocardioidaceae_	H11	3.67	3.48	0.03
Bacteria; Firmicutes; Negativicutes; Selenomonadales; Sporomusaceae; Anaerosporomusa	C11	3.63	3.50	0.03
Bacteria; Firmicutes; Clostridia; Eubacteriales; Oscillospiraceae; Sporobacter	C11	3.53	3.46	0.04
Bacteria; Firmicutes; Clostridia; Eubacteriales; Clostridiaceae; Clostridium; _Clostridium_	H16	5.41	5.02	0.04
Bacteria; Firmicutes; Clostridia; Eubacteriales; Clostridiaceae; Oxobacter; Oxobacterpfennigii	C16	4.82	4.39	0.04
Bacteria; Firmicutes; Clostridia; Eubacteriales; Lachnospiraceae; _Lachnospiraceae; _Lachnospiraceae_	M16	4.43	4.00	0.04
Bacteria; Firmicutes; Clostridia; Eubacteriales; Clostridiaceae; Fonticella; Fonticellatunisiensis	C16	4.18	3.86	0.03

Bacteria; Firmicutes; Bacilli; Bacillales; Paenibacillaceae; Paenibacillus; _Paenibacillus_	C16	4.12	3.81	0.03
Bacteria; Proteobacteria; Alphaproteobacteria; Hyphomicrobiales; Bradyrhizobiaceae; _Bradyrhizobiaceae_; _Bradyrhizobiaceae_	H16	3.88	3.62	0.03
Bacteria; Firmicutes; Clostridia; Eubacteriales; Clostridiaceae; Clostridium; Clostridiumbotulinum	H16	3.79	3.81	0.04
Bacteria; Firmicutes; Bacilli; Bacillales; Paenibacillaceae; _Paenibacillaceae; _Paenibacillaceae_	C16	3.81	3.68	0.03
Bacteria; Firmicutes; Clostridia; Eubacteriales; Clostridiaceae; Clostridium	H16	5.45	5.02	0.04
Bacteria; Firmicutes; Clostridia; Eubacteriales; Clostridiaceae; Oxobacter	C16	4.82	4.40	0.04
Bacteria; Firmicutes; Clostridia; Eubacteriales; Lachnospiraceae	M16	4.58	4.08	0.03
Bacteria; Firmicutes; Clostridia; Eubacteriales; Lachnospiraceae; _Lachnospiraceae_	M16	4.43	3.98	0.04
Bacteria; Firmicutes; Clostridia; Eubacteriales; Clostridiaceae; Fonticella	C16	4.18	3.96	0.03
Bacteria; Firmicutes; Bacilli; Bacillales; Paenibacillaceae	C16	4.29	3.97	0.03
Bacteria; Firmicutes; Bacilli; Bacillales; Paenibacillaceae; Paenibacillus	C16	4.12	3.81	0.03
Bacteria; Proteobacteria; Alphaproteobacteria	H16	3.88	3.58	0.03
Bacteria; Proteobacteria; Alphaproteobacteria; Hyphomicrobiales	H16	3.88	3.64	0.03
Bacteria; Proteobacteria; Alphaproteobacteria; Hyphomicrobiales; Bradyrhizobiaceae	H16	3.88	3.63	0.03
Bacteria; Proteobacteria; Alphaproteobacteria; Hyphomicrobiales; Bradyrhizobiaceae; _Bradyrhizobiaceae_	H16	3.88	3.61	0.03
Bacteria; Firmicutes; Bacilli; Bacillales; Paenibacillaceae; _Paenibacillaceae_	C16	3.81	3.78	0.03
Bacteria; Firmicutes; Clostridia; Clostridiales; Clostridiaceae1; Clostridium sensustricto10	H23	5.21	4.51	0.04
Bacteria; Firmicutes; Clostridia; Clostridiales; Clostridiaceae1; Clostridium sensustricto10; uncultured bacterium	H23	4.98	4.28	0.03
Bacteria; Firmicutes; Clostridia; Clostridiales; Clostridiaceae1; Clostridium sensustricto10_Ambiguous_taxa	H23	4.90	4.15	0.03
Bacteria; Firmicutes; Clostridia; Clostridiales; Clostridiaceae1; Clostridium sensustricto8; uncultured bacterium	H23	4.90	4.15	0.03
Bacteria; Firmicutes; Clostridia; Clostridiales; Clostridiaceae1; Clostridium sensustricto8	H23	4.89	4.22	0.04

Archaea; Euryarchaeota; Methanomicrobia; Methanocellales; Methanocellaceae; Methanocella; uncultured archaeon	H23	4.74	3.73	0.03
Bacteria; Firmicutes; Clostridia; Clostridiales; Clostridiaceae1; Clostridium sensustricto18	H23	5.09	4.77	0.03
Bacteria; Firmicutes; Clostridia; Clostridiales; FamilyXVIII; uncultured; uncultured bacterium	C23	4.76	4.44	0.03
Bacteria; Firmicutes; Clostridia; Clostridiales; Clostridiaceae1; Clostridium sensustrictotricto8_Ambiguous_taxa	H23	4.63	3.96	0.03
Bacteria; Actinobacteria; Actinobacteria; Propionibacteriales; Nocardioidaceae; Nocardioides	H23	4.56	3.67	0.03
Bacteria; Ignavibacteriae; Ignavibacteria; Ignavibacteriales; BSV40	C23	4.58	4.01	0.04
Bacteria; Firmicutes; Clostridia; Clostridiales; Ruminococcaceae; Ruminiclostridium1; uncultured bacterium	C23	4.58	3.91	0.03
Bacteria; Firmicutes; Clostridia; Clostridiales; Clostridiaceae1; Clostridium sensustrictotricto18; Clostridium sporogenes	H23	4.82	4.53	0.03
Bacteria; Acidobacteria; Solibacteres; Solibacterales; Solibacteraceae_Subgroup3_; Candidatus Solibacter	C23	4.39	3.76	0.03
Bacteria; Proteobacteria; Deltaproteobacteria; Myxococcales; Polyangiaceae; Sorangium; uncultured bacterium	H23	4.34	3.70	0.04
Bacteria; Actinobacteria; Thermoleophilia; Gaiellales; uncultured; uncultured bacterium; uncultured bacterium	H23	4.32	3.64	0.04
Bacteria; Firmicutes; Clostridia; Clostridiales; Heliobacteriaceae; Hydrogenispora; uncultured bacterium	C23	4.34	3.74	0.04
Bacteria; Firmicutes; Clostridia; Clostridiales; Lachnospiraceae; Anaerosporobacter	H23	4.34	3.83	0.03
Bacteria; Firmicutes; Clostridia; Clostridiales; Peptococcaceae; uncultured; uncultured bacterium	C23	4.28	3.69	0.03
Bacteria; Firmicutes; Clostridia; Clostridiales; Ruminococcaceae; RuminococcaceaeUCG_010; uncultured bacterium	C23	4.24	3.71	0.03
Bacteria; Acidobacteria; Acidobacteria; Acidobacteriales; Acidobacteriaceae_Subgroup1_; uncultured	C23	4.22	3.68	0.03
Bacteria; Firmicutes; Clostridia; Clostridiales; Christensenellaceae; uncultured; uncultured bacterium	M23	4.18	3.61	0.04
Bacteria; Acidobacteria; Solibacteres; Solibacterales; Solibacteraceae_Subgroup3_; Candidatus Solibacter; uncultured bacterium	C23	4.16	3.55	0.04
Bacteria; Firmicutes; Clostridia; Clostridiales; Clostridiaceae1; Fonticella_Ambiguous_taxa	C23	4.13	3.47	0.03
Bacteria; Firmicutes; Clostridia; Clostridiales; Clostridiaceae1; Fonticella	C23	4.15	3.64	0.03
Bacteria; Proteobacteria; Deltaproteobacteria; Myxococcales; Polyangiaceae	H23	4.13	3.62	0.03

Bacteria; Cyanobacteria; ML635J_21; uncultured bacterium; uncultured bacterium; uncultured bacterium; uncultured bacterium	H23	4.12	3.53	0.04
Bacteria; Actinobacteria; Actinobacteria; Propionibacteriales; Nocardioidaceae; Nocardioides; uncultured bacterium	H23	4.07	3.37	0.03
Bacteria; Proteobacteria; Alphaproteobacteria; Rhizobiales; Bradyrhizobiaceae	H23	4.11	3.53	0.03
Bacteria; Firmicutes; Clostridia; Clostridiales; Christensenellaceae; uncultured	M23	4.05	3.42	0.03
Bacteria; Firmicutes; Clostridia; Clostridiales; Clostridiaceae1; Clostridium sensustricto9; uncultured bacterium	H23	4.04	3.49	0.03
Archaea; Euryarchaeota; Methanomicrobia; Methanocellales; Methanocellaceae; RiceClusterI; uncultured archaeon	H23	3.98	3.33	0.03
Bacteria; Proteobacteria; Deltaproteobacteria; Myxococcales; Sandaracinaceae; uncultured; uncultured bacterium	H23	3.99	3.27	0.03
Bacteria; Firmicutes; Bacilli; Bacillales; Paenibacillaceae; Paenibacillus	H23	3.98	3.44	0.03
Bacteria; Firmicutes; Clostridia; Clostridiales; Lachnospiraceae; Anaerostipes; uncultured bacterium	H23	4.05	3.64	0.03
Bacteria; Actinobacteria; Thermoleophilia; Solirubrobacterales	H23	3.93	3.37	0.03
Bacteria; Proteobacteria; Deltaproteobacteria; Desulfuromonadales; Geobacteraceae; Geobacter; uncultured bacterium	M23	4.06	3.61	0.03
Bacteria; Firmicutes; Clostridia; Clostridiales; Peptococcaceae; Desulfotomaculum	C23	3.90	3.24	0.03
Bacteria; Chloroflexi; Anaerolineae; Anaerolineales; Anaerolineaceae; uncultured; uncultured bacterium	M23	3.92	3.40	0.03
Bacteria; Firmicutes; Clostridia; Clostridiales; Clostridiaceae1; Clostridium sensustricto9	H23	3.90	3.36	0.04
Bacteria; Proteobacteria; Deltaproteobacteria; Myxococcales; Polyangiaceae; Sorangium	H23	3.86	3.27	0.03
Bacteria; Elusimicrobia; Elusimicrobia; LineageI; UnknownFamily; Candidatus Endomicrobium; uncultured bacterium	C23	3.94	3.53	0.03
Bacteria; Firmicutes; Clostridia; Clostridiales; Peptococcaceae; Sporotomaculum; uncultured bacterium	C23	3.88	3.46	0.03
Bacteria; Proteobacteria; Deltaproteobacteria; Bdellovibrionales; Bdellovibrionaceae; OM27clade; uncultured bacterium	H23	3.82	3.36	0.04
Archaea; Euryarchaeota; Methanomicrobia; Methanocellales; Methanocellaceae; Methanocella	H23	4.74	3.76	0.03
Bacteria; Firmicutes; Clostridia; Clostridiales; FamilyXVIII	C23	4.76	4.44	0.03
Bacteria; Firmicutes; Clostridia; Clostridiales; FamilyXVIII; uncultured	C23	4.76	4.43	0.03

Bacteria; Actinobacteria; Actinobacteria; Propionibacteriales	H23	4.80	3.88	0.04
Bacteria; Actinobacteria; Actinobacteria; Propionibacteriales; Nocardioidaceae	H23	4.80	3.97	0.04
Bacteria; Ignavibacteriae	C23	4.73	4.14	0.04
Bacteria; Ignavibacteriae; Ignavibacteria	C23	4.73	4.05	0.04
Bacteria; Ignavibacteriae; Ignavibacteria; Ignavibacteriales	C23	4.73	4.08	0.04
Bacteria; Acidobacteria; Solibacteres	C23	4.59	3.91	0.03
Bacteria; Acidobacteria; Solibacteres; Solibacterales	C23	4.59	3.91	0.03
Bacteria; Acidobacteria; Solibacteres; Solibacterales; Solibacteraceae_Subgroup3_	C23	4.59	3.88	0.03
Bacteria; Actinobacteria; Thermoleophilia	H23	4.47	3.77	0.04
Bacteria; Actinobacteria; Thermoleophilia; Gaiellales	H23	4.32	3.64	0.04
Bacteria; Actinobacteria; Thermoleophilia; Gaiellales; uncultured	H23	4.32	3.71	0.04
Bacteria; Actinobacteria; Thermoleophilia; Gaiellales; uncultured; uncultured bacterium	H23	4.32	3.77	0.04
Bacteria; Firmicutes; Clostridia; Clostridiales; Heliobacteriaceae	C23	4.34	3.80	0.04
Bacteria; Firmicutes; Clostridia; Clostridiales; Heliobacteriaceae; Hydrogenispora	C23	4.34	3.84	0.04
Bacteria; Firmicutes; Clostridia; Clostridiales; Peptococcaceae; uncultured	C23	4.28	3.69	0.03
Bacteria; Firmicutes; Clostridia; Clostridiales; Ruminococcaceae; Ruminococcaceae UCG_010	C23	4.24	3.70	0.03
Bacteria; Acidobacteria; Acidobacteria	C23	4.22	3.65	0.03
Bacteria; Acidobacteria; Acidobacteria; Acidobacteriales	C23	4.22	3.64	0.03
Bacteria; Acidobacteria; Acidobacteria; Acidobacteriales; Acidobacteriaceae_Subgroup1_	C23	4.22	3.71	0.03
Bacteria; Cyanobacteria; ML635J_21	H23	4.12	3.55	0.04
Bacteria; Cyanobacteria; ML635J_21; uncultured bacterium	H23	4.12	3.58	0.04

Bacteria; Cyanobacteria; ML635J_21; uncultured bacterium; uncultured bacterium	H23	4.12	3.50	0.04
Bacteria; Cyanobacteria; ML635J_21; uncultured bacterium; uncultured bacterium; uncultured bacterium	H23	4.12	3.54	0.04
Bacteria; Proteobacteria; Deltaproteobacteria; Myxococcales; Sandaracinaceae	H23	3.99	3.39	0.03
Bacteria; Proteobacteria; Deltaproteobacteria; Myxococcales; Sandaracinaceae; uncultured	H23	3.99	3.48	0.03
Bacteria; Firmicutes; Clostridia; Clostridiales; Lachnospiraceae; Anaerostipes	H23	4.05	3.64	0.03
Bacteria; Proteobacteria; Deltaproteobacteria; Desulfuromonadales	M23	4.06	3.69	0.03
Bacteria; Proteobacteria; Deltaproteobacteria; Desulfuromonadales; Geobacteraceae	M23	4.06	3.63	0.03
Bacteria; Proteobacteria; Deltaproteobacteria; Desulfuromonadales; Geobacteraceae; Geobacter	M23	4.06	3.65	0.03
Bacteria; Chloroflexi; Anaerolineae	M23	3.92	3.25	0.03
Bacteria; Chloroflexi; Anaerolineae; Anaerolineales	M23	3.92	3.31	0.03
Bacteria; Chloroflexi; Anaerolineae; Anaerolineales; Anaerolineaceae	M23	3.92	3.57	0.03
Bacteria; Chloroflexi; Anaerolineae; Anaerolineales; Anaerolineaceae; uncultured	M23	3.92	3.30	0.03
Bacteria; Elusimicrobia	C23	3.94	3.50	0.03
Bacteria; Elusimicrobia; Elusimicrobia	C23	3.94	3.47	0.03
Bacteria; Elusimicrobia; Elusimicrobia; LineageI	C23	3.94	3.54	0.03
Bacteria; Elusimicrobia; Elusimicrobia; LineageI; UnknownFamily	C23	3.94	3.53	0.03
Bacteria; Elusimicrobia; Elusimicrobia; LineageI; UnknownFamily; Candidatus Endomicrobium	C23	3.94	3.50	0.03
Bacteria; Firmicutes; Clostridia; Clostridiales; Peptococcaceae; Sporotomaculum	C23	3.88	3.40	0.03
Bacteria; Proteobacteria; Deltaproteobacteria; Bdellovibrionales	H23	3.82	3.60	0.04
Bacteria; Proteobacteria; Deltaproteobacteria; Bdellovibrionales; Bdellovibrionaceae	H23	3.82	3.40	0.04
Bacteria; Proteobacteria; Deltaproteobacteria; Bdellovibrionales; Bdellovibrionaceae; OM27clade	H23	3.82	3.29	0.04

Bacteria; Firmicutes; Clostridia; Eubacteriales; Clostridiaceae; Clostridium; _Clostridium_	H30	5.40	4.94	0.04
Bacteria; Firmicutes; Clostridia; Eubacteriales; Clostridiaceae; Thermobrachium; Thermobrachiumcelere	M30	4.90	4.41	0.03
Bacteria; Firmicutes; Clostridia; Eubacteriales; Clostridiaceae; Oxobacter; Oxobacterpfennigii	C30	4.95	4.61	0.03
Archaea; Euryarchaeota; Methanomicrobia; Methanocellales; Methanocellaceae; Methanocella; Methanocellasp_PtaU1_Bin125	C30	4.53	3.96	0.03
Bacteria; Firmicutes; Clostridia; Eubacteriales; Clostridiaceae; Clostridium; Clostridiumbotulinum	H30	4.04	3.76	0.03
Archaea; Euryarchaeota; Methanomicrobia; Methanocellales; Methanocellaceae; Methanocella; Methanocellaconradii	C30	3.92	3.65	0.03
Bacteria; Firmicutes; Clostridia; Eubacteriales; Clostridiaceae; Clostridium; Clostridiumsporogenes	H30	4.03	3.77	0.03
Bacteria; Firmicutes; Bacilli; Bacillales; Bacillaceae; Neobacillus; Neobacillusvireti	M30	4.06	3.81	0.03
Bacteria; Firmicutes; Clostridia; Eubacteriales; Clostridiaceae; Clostridium; Clostridiumcavendishii	C30	4.16	3.94	0.04
Bacteria; Firmicutes; Bacilli; Bacillales; Bacillaceae; Bacillus; _Bacillus_	C30	3.69	3.58	0.03
Bacteria; Firmicutes; Clostridia; Eubacteriales; Clostridiaceae; Clostridium	H30	5.50	4.99	0.03
Bacteria; Firmicutes; Clostridia; Eubacteriales; Clostridiaceae; Thermobrachium	M30	4.90	4.43	0.03
Bacteria; Firmicutes; Clostridia; Eubacteriales; Clostridiaceae; Oxobacter	C30	4.95	4.60	0.03
Archaea; Euryarchaeota; Methanobacteria	M30	4.59	4.00	0.04
Archaea; Euryarchaeota; Methanobacteria; Methanobacteriales	M30	4.59	4.09	0.04
Archaea; Euryarchaeota; Methanobacteria; Methanobacteriales; Methanobacteriaceae	M30	4.59	4.04	0.04
Archaea; Euryarchaeota; Methanobacteria; Methanobacteriales; Methanobacteriaceae; Methanobacterium	M30	4.59	4.09	0.04
Bacteria; Firmicutes; Clostridia; Eubacteriales; Peptococcaceae	C30	4.83	4.16	0.04
Bacteria; Firmicutes; Clostridia; Eubacteriales; Peptococcaceae; Pelotomaculum	C30	4.51	4.03	0.04
Bacteria; Firmicutes; Bacilli; Bacillales; Bacillaceae; Neobacillus	M30	4.06	3.8	0.03

Table S7 Taxonomic composition of transcripts affiliated to specific metabolic pathways as shown in Figure 3. The values show the average relative mRNA abundance of the transcriptionally most active families within each metabolic pathway at the control treatment (0.00%), as well as at moderate (1.75%) and high (3.50%) salinity. Each value represents the average metatranscriptomic abundance calculated across the triplicate samples of all four preincubation times (n = 12).

pathway	family	Salinity		
		0.00%	1.75%	3.50%
CAZyme	Bacillaceae	0.25	0.26	0.16
CAZyme	Burkholderiaceae	0.11	0.10	0.06
CAZyme	Clostridiaceae	0.32	0.58	1.74
CAZyme	Enterobacteriaceae	0.29	0.23	0.15
CAZyme	Flavobacteriaceae	0.28	0.23	0.17
CAZyme	Francisellaceae	0.09	0.13	0.09
CAZyme	Lachnospiraceae	0.04	0.09	0.04
CAZyme	Microbacteriaceae	0.09	0.08	0.02
CAZyme	Micrococcaceae	0.11	0.11	0.10
CAZyme	Paenibacillaceae	0.03	0.04	0.01
CAZyme	Peptococcaceae	0.13	0.17	0.11
CAZyme	Pseudomonadaceae	0.19	0.25	0.21

CAZyme	Streptomycetaceae	0.06	0.10	0.07
CAZyme	Thermoanaerobacteraceae	0.00	0.00	0.00
Glycolysis	Christensenellaceae	0.18	0.41	0.11
Glycolysis	Clostridiaceae	2.57	7.72	42.02
Glycolysis	Lachnospiraceae	0.18	1.13	0.38
Glycolysis	Oscillospiraceae	1.39	1.70	0.27
Glycolysis	Peptococcaceae	1.42	0.47	0.14
Glycolysis	Symbiobacteriaceae	0.65	0.89	0.02
Pyruvate metabolism	Clostridiaceae	1.46	4.37	18.95
Pyruvate metabolism	Desulfallaceae	1.52	0.88	0.28
Pyruvate metabolism	Lachnospiraceae	0.32	1.04	0.24
Pyruvate metabolism	Oscillospiraceae	1.54	1.92	0.28
Pyruvate metabolism	Peptococcaceae	1.41	0.82	0.46
Pyruvate metabolism	Pseudomonadaceae	0.37	0.21	0.82
Ethanol fermentation	Bacillaceae	0.25	0.29	0.46
Ethanol fermentation	Christensenellaceae	0.43	0.30	0.38
Ethanol fermentation	Clostridiaceae	2.54	13.65	48.74
Ethanol fermentation	Lachnospiraceae	0.30	0.96	0.00
Ethanol fermentation	Oscillospiraceae	2.37	0.82	0.51
Ethanol fermentation	Peptococcaceae	2.73	0.81	0.14
Acetatogenesis	Bacillaceae	0.20	0.27	0.35

Acetatogenesis	Christensenellaceae	0.32	0.24	0.45
Acetatogenesis	Clostridiaceae	2.01	11.94	40.39
Acetatogenesis	Lachnospiraceae	0.32	1.05	0.00
Acetatogenesis	Oscillospiraceae	2.04	0.72	0.37
Acetatogenesis	Peptococcaceae	2.16	0.56	0.12
Propionate fermentation	Bacillaceae	0.00	0.81	0.64
Propionate fermentation	Clostridiaceae	0.00	0.51	0.98
Propionate fermentation	Eggerthellaceae	0.39	0.68	0.15
Propionate fermentation	Nitrospiraceae	0.80	0.00	0.15
Propionate fermentation	Peptococcaceae	5.51	0.26	0.83
Propionate fermentation	Symbiobacteriaceae	2.27	0.00	0.00
Acetoclastic methanogenesis	Methanobacteriales	0.34	0.73	1.39
Acetoclastic methanogenesis	Methanocellales	2.29	2.26	1.58
Acetoclastic methanogenesis	Methanomassiliicoccales	0.20	0.11	0.21
Acetoclastic methanogenesis	Methanomicrobiales	0.00	0.29	0.04
Acetoclastic methanogenesis	Methanosarcinales	64.78	63.56	67.35
Acetoclastic methanogenesis	Methanotrichales	0.00	0.20	0.11
Hydrogenotrophic methanogenesis	Methanobacteriales	5.80	20.92	7.32
Hydrogenotrophic methanogenesis	Methanocellales	74.69	47.71	59.28
Hydrogenotrophic methanogenesis	Methanomicrobiales	0.63	0.00	0.86
Hydrogenotrophic methanogenesis	Methanopyrales	0.00	0.00	0.14

Hydrogenotrophic methanogenesis	Methanosarcinales	6.95	13.09	14.40
Hydrogenotrophic methanogenesis	Methanotrichales	0.16	0.00	0.10
Methylotrophic methanogenesis	Methanobacteriales	1.64	1.41	1.64
Methylotrophic methanogenesis	Methanomassiliicoccales	24.12	24.73	27.94
Methylotrophic methanogenesis	Methanosarcinales	41.36	46.65	48.39

Table S8 Percentage and total number of mRNA reads with search hits in the CAZy database, related to the decomposition of cellulose, chitin, xylan, pectin, and others. The sample identifiers C, M, and H represent the control (0.00%) treatment, as well as moderate (1.75%), and high (3.50%) salinity, respectively. The first two digits of the sample number indicate the total incubation period, including preincubation time and salinity exposure (days 9+2 [11]; 14+2 [16]; 21+2 [23]; 28+2 [30]). The last digit distinguishs between the triplicate samples of a given treatment.

Sample ID	No. of sequences with hits in CAZyme	No. of sequences	% CAZyme	Cellulose	Chitin	Xylan	Pectin	Others (Hemicellulose)
C111	3042	87712	3.47	266	624	506	39	625
C112	2472	58168	4.25	269	532	471	28	533
C113	2942	73059	4.03	327	584	581	53	649
C161	2279	48789	4.67	211	487	397	31	476
C162	2478	49499	5.01	240	525	441	29	528
C163	2451	50320	4.87	272	538	412	36	511
C231	2860	54539	5.24	363	597	525	38	578
C232	2476	45537	5.44	295	501	434	43	477
C233	3277	68482	4.79	356	736	631	56	678
C301	1984	55006	3.61	215	412	312	29	416
C302	1754	51478	3.41	182	373	301	27	350
C303	2433	65150	3.73	235	574	412	24	492

M111	4049	79534	5.09	281	790	523	37	785
M112	2823	51450	5.49	202	546	359	37	516
M113	2867	52440	5.47	181	483	401	33	545
M161	2361	47072	5.02	156	448	332	23	442
M162	1833	38706	4.74	119	316	239	19	361
M163	2243	49843	4.50	181	414	344	33	463
M231	1881	37454	5.02	140	372	262	17	395
M232	1804	43586	4.14	119	300	251	28	382
M233	2276	47775	4.76	133	422	322	35	437
M301	2310	62226	3.71	171	438	339	41	453
M302	2599	66328	3.92	189	507	378	35	448
M303	1385	47202	2.93	91	274	201	21	271
H111	2165	58583	3.70	129	289	232	44	433
H112	1491	36313	4.11	85	237	148	26	300
H113	1601	41268	3.88	106	245	183	26	298
H161	1415	47734	2.96	90	228	232	22	272
H162	1156	41692	2.77	87	192	159	18	267
H163	901	36507	2.47	55	161	119	11	192
H231	1388	45958	3.02	94	226	194	49	278
H232	1758	62606	2.81	95	332	263	74	327
H233	1044	36580	2.85	77	181	170	32	214

H301	1849	77107	2.40	156	311	349	71	353
H302	885	41329	2.14	47	191	140	22	147
H303	1766	75264	2.35	127	305	258	32	363

Table S9 Adaptive salinity response features. The values show the average relative mRNA abundance of particular stress response mechanisms at the control treatment (0.00%), as well as at moderate (1.75%) and high (3.50%) salinity. Each value represents the average metatranscriptomic abundance calculated across the triplicate samples of all four preincubation times (n = 12).

representative name	symbol	database	Level_1	Level_2	Level_3	Level_4	ID	Salinity		
								0.00%	1.75%	3.50%
flagellin	fliC, hag	KEGG	Environmental Information Processing	Signal transduction	ko02020 Two-component system	K02406 flagellin	ID2406	0.28	0.19	0.45
Stress Response: Osmotic stress	-	SEED	Stress Response, Defense and Virulence	Stress Response, Defense and Virulence	Stress Response: Osmotic stress	Betaine biosynthesis from glycine	ID16567	0.00	0.00	0.00
Stress Response: Osmotic stress	-	SEED	Stress Response, Defense and Virulence	Stress Response, Defense and Virulence	Stress Response: Osmotic stress	Choline uptake and conversion to betaine clusters (Opu family)	ID23088	0.03	2.25	0.72
Stress Response: Osmotic stress	-	SEED	Stress Response, Defense and Virulence	Stress Response, Defense and Virulence	Stress Response: Osmotic stress	Ectoine synthesis	ID23226	0.00	0.00	0.00
Stress Response: Osmotic stress	-	SEED	Stress Response, Defense and Virulence	Stress Response, Defense and Virulence	Stress Response: Osmotic stress	Ectoine, hydroxyectoine uptake and catabolism	ID23239	0.00	0.00	0.00
Stress Response: Osmotic stress	-	SEED	Stress Response, Defense and Virulence	Stress Response, Defense and Virulence	Stress Response: Osmotic stress	EnvZ and OmpR regulon	ID23140	0.00	0.00	0.01
Stress Response:	-	SEED	Stress Response,	Stress Response,	Stress Response:	Glycine betaine synthesis from	ID23	0.0	0.0	0.0

Osmotic stress			Defense and Virulence	Defense and Virulence	Osmotic stress	choline	089	1	1	1
Stress Response: Osmotic stress	-	SEED	Stress Response, Defense and Virulence	Stress Response, Defense and Virulence	Stress Response: Osmotic stress	Mannosylglucosylglycerate synthesis	ID23 427	0.0 0	0.0 0	0.0 0
Stress Response: Osmotic stress	-	SEED	Stress Response, Defense and Virulence	Stress Response, Defense and Virulence	Stress Response: Osmotic stress	Osmoregulation (Osmotically inducible protein OsmY)	ID14 795	0.1 0	0.0 3	0.0 8
V/A-type H+/Na+-transporting ATPase	ATPVG, ahaH, atpH	KEGG	Metabolism	Energy metabolism	ko00190 Oxidative phosphorylation	K02107 V/A-type H+/Na+-transporting ATPase subunit G/H	ID21 07	0.0 3	0.0 2	0.0 2
V/A-type H+/Na+-transporting ATPase	ATPVA, ntpA, atpA	KEGG	Metabolism	Energy metabolism	ko00190 Oxidative phosphorylation	K02117 V/A-type H+/Na+-transporting ATPase subunit A	ID21 17	0.1 4	0.1 3	0.2 0
V/A-type H+/Na+-transporting ATPase	ATPVB, ntpB, atpB	KEGG	Metabolism	Energy metabolism	ko00190 Oxidative phosphorylation	K02118 V/A-type H+/Na+-transporting ATPase subunit B	ID21 18	0.1 1	0.1 2	0.1 7
V/A-type H+/Na+-transporting ATPase	ATPVC, ntpC, atpC	KEGG	Metabolism	Energy metabolism	ko00190 Oxidative phosphorylation	K02119 V/A-type H+/Na+-transporting ATPase subunit C	ID21 19	0.0 7	0.0 6	0.0 6
V/A-type H+/Na+-transporting ATPase	ATPVD, ntpD, atpD	KEGG	Metabolism	Energy metabolism	ko00190 Oxidative phosphorylation	K02120 V/A-type H+/Na+-transporting ATPase subunit D	ID21 20	0.0 6	0.0 5	0.0 7
V/A-type H+/Na+-	ATPVE,	KEGG	Metabolism	Energy metabolism	ko00190 Oxidative	K02121 V/A-type H+/Na+-	ID21	0.0	0.0	0.0

transporting ATPase	ntpE, atpE				phosphorylation	transporting ATPase subunit E	21	3	2	3
V/A-type H+/Na+-transporting ATPase	ATPVF, ntpF, atpF	KEGG	Metabolism	Energy metabolism	ko00190 Oxidative phosphorylation	K02122 V/A-type H+/Na+-transporting ATPase subunit F	ID2122	0.02	0.02	0.02
V/A-type H+/Na+-transporting ATPase	ATPVI, ntpI, atpI	KEGG	Metabolism	Energy metabolism	ko00190 Oxidative phosphorylation	K02123 V/A-type H+/Na+-transporting ATPase subunit I	ID2123	0.17	0.12	0.13
V/A-type H+/Na+-transporting ATPase	ATPVI, ntpI, atpI	KEGG	Metabolism	Energy metabolism	ko00190 Oxidative phosphorylation	K02124 V/A-type H+/Na+-transporting ATPase subunit K	ID2124	0.05	0.04	0.05

Supplemental Methods

RNA extraction, library preparation, and sequencing

Total RNA was extracted from slurries using a previously established method (1, 2). Fresh soil (0.5 g) was mixed with the same amount of glass beads and suspended in 700 µl of TPM buffer (0.5 M Tris pH 7.0, 1.7% polyvinylpyrrolidone, 0.2 M $MgCl_2$). The mixture was shaken at 6.0 m s^{-1} for 45 s followed by centrifugation at 20,000 × *g* for 4 min. The pellet was resuspended in 700 µl PBL buffer (0.05 M Tris pH 7.0, 0.05 M Na_2EDTA, 0.1% SDS w/v, 6% v/v phenol) and the lysis procedure was repeated as described above. The supernatants of the two lysis procedure rounds were combined and purified by two-step phase extraction with phenol-chloroform-isoamylalcohol and chloroform-isoamylalcohol. RNA was precipitated with pre-cooled isopropanol and resuspended in 50 µl of TE buffer (10 mM Tris-HCl, 1 mM EDTA [pH 8.0]). RNA extracts were treated with DNase I (Ambion, Austin, TX, USA) and purified using the RNA Clean and Concentrator kit (ZymoResearch, Irvine, CA, USA) according to the manufacturer's instructions. This also removed non-coding RNA. The integrity of the purified RNA was checked by Bio-Rad Experion™ and RNA HighSens Chips (Bio-Rad, Hercules, CA, USA).

A total of 36 samples of total RNA (3 replicate microcosms × 4 time points × 3 salt treatments [0, 1.75%, and 3.50% salinity]) were subjected to cDNA synthesis using the NEBNext® Ultra™ Directional RNA Library Prep Kit for Illumina® (New England Biolabs, Ipswich, MA, USA) according to the manufacturer's instructions. cDNA yield and integrity were determined by Qubit® (ThermoFisher Scientific) and automated high-resolution electrophoresis (Experion, Bio-Rad, Hercules, CA, USA). The 36 cDNA libraries were sequenced on an Illumina HiSeq platform in paired-end

mode (2 × 250 bp) at the Max Planck Genome Centre Cologne. Illumina RNA-Seq resulted in 256,826,625 reads, ranging from 3,678,228 to 15,343,424 reads per cDNA library (Table S1).

Bioinformatics

Analysis of total RNA reads was carried out with a customized pipeline as described previously (1, 2). Briefly, the adaptor sequences of the raw Illumina paired-end reads were trimmed using Cutadapt (3) with a minimum mean quality score of 20. Both 16S and 18S rRNA reads were extracted from the metatranscriptomic data sets using SortMeRNA 2.0 (4) with SILVA (release 138). A total of 91,123,349 quality-filtered reads were obtained, of which 39,940,752 and 1,932,296 reads were derived from 16S rRNA and mRNA, respectively (Table S1). USEARCH7 and QIIME 2 (5) were used as the overall framework for analysis of the 16S rRNA-derived reads (1). The 16S rRNA reads were clustered using 97% sequence identity as the cutoff (6). All OTUs assigned to a particular family were retained for further analysis.

The taxonomic assignment and functional annotation of the mRNA reads were carried out using the UBLAST algorithm implemented in USEARCH7 applying an e-value cutoff of $1e^{-5}$, maxhits 50, and maxaccepts 50 for database searches against the NCBI non-redundant protein database (release 2023). MEGAN6 Ultimate Edition (7). was used for parsing and downstream analysis of the UBLAST output. Taxonomy assignments were made using the “lowest common ancestor” (LCA) method with the following parameters: minimum support 1, minimum bit score 150, top percent 10. Taxonomically assigned mRNA was functionally annotated according to KEGG categories and SEED subsystems implemented in MEGAN6 Ultimate Edition (7-9). In-depth sub-transcriptome analysis was conducted using in-house scripts, with mRNA reads related to particular functional genes and pathways being extracted from

MEGAN6 Ultimate Edition and blasted against the NCBI non-redundant protein database. The custom module definitions were derived from the KEGG and SEED modules (Table S2) (10).

Transcript analysis of CAZyme genes

The annotation of carbohydrate-active enzyme (CAZyme) encoding transcripts was achieved by querying putative mRNA data sets against dbCAN2 (11) using DIAMOND with the default e-value cutoff of 1e-5. Functional annotation of CAZyme modules such as, for example, those involved in cellulose or xylan degradation, was done by querying the sequences using a custom Python script against the dbCAN mapping file (1). A mapping file for functional annotation was created using all available entries in dbCAN. Subsets of mRNA reads linked to CAZyme functions were taxonomically assigned using custom Python scripts (12) with prokaryotic genomes from NCBI RefSeq and NCBI nr serving as reference databases using Kraken2 (11).

References

1. Peng J, Wegner C-E, Bei Q, Liu P, Liesack W. 2018. Metatranscriptomics reveals a differential temperature effect on the structural and functional organization of the anaerobic food web in rice field soil. Microbiome 6:1-16.
2. Li X, Bei Q, Rabiei Nematabad M, Peng J, Liesack W. 2024a. Time-shifted expression of acetoclastic and methylotrophic methanogenesis by a single Methanosarcina genomospecies predominates the methanogen dynamics in Philippine rice field soil. Microbiome 12:39.

3. Martin M. 2011. Cutadapt removes adapter sequences from high-throughput sequencing reads. EMBnet journal 17:10-12.

4. Kopylova E, Noé L, Touzet H. 2012. SortMeRNA: fast and accurate filtering of ribosomal RNAs in metatranscriptomic data. Bioinformatics 28:3211-3217.

5. Bolyen E, Rideout JR, Dillon MR, Bokulich NA, Abnet CC, Al-Ghalith GA, Alexander H, Alm EJ, Arumugam M, Asnicar F. 2019. Reproducible, interactive, scalable and extensible microbiome data science using QIIME 2. Nat Biotechnol 37:852-857.

6. Santos-Medellín C, Liechty Z, Edwards J, Nguyen B, Huang B, Weimer BC, Sundaresan V. 2021. Prolonged drought imparts lasting compositional changes to the rice root microbiome. Nat Plants 7:1065-1077.

7. Huson DH, Auch AF, Qi J, Schuster SC. 2007. MEGAN analysis of metagenomic data. Genome Res 17:377-386.

8. Kanehisa M, Goto S. 2000. KEGG: kyoto encyclopedia of genes and genomes. Nucleic Acids Res 28:27-30.

9. Overbeek R, Begley T, Butler RM, Choudhuri JV, Chuang H-Y, Cohoon M, de Crécy-Lagard V, Diaz N, Disz T, Edwards R. 2005. The subsystems approach to genome annotation and its use in the project to annotate 1000 genomes. Nucleic Acids Res 33:5691-5702.

10. Woodcroft BJ, Singleton CM, Boyd JA, Evans PN, Emerson JB, Zayed AA, Hoelzle RD, Lamberton TO, McCalley CK, Hodgkins SB. 2018. Genome-centric view of carbon processing in thawing permafrost. Nature 560:49-54.

11. Yin Y, Mao X, Yang J, Chen X, Mao F, Xu Y. 2012. dbCAN: a web resource for

automated carbohydrate-active enzyme annotation. Nucleic Acids Res 40:W445-W451.

12. Menzel P, Ng KL, Krogh A. 2016. Fast and sensitive taxonomic classification for metagenomics with Kaiju. Nat Commun 7:11257.
